# Vigi4Eudra-score: Evaluation of the completeness of spontaneous adverse drug reaction reports in EudraVigilance

**DOI:** 10.1371/journal.pone.0343694

**Published:** 2026-02-25

**Authors:** Patrick Christ, Diana Dubrall, Dennis Lex, Thomas Grüger, Matthias Schmid, Bernhardt Sachs

**Affiliations:** 1 Federal Institute for Drugs and Medical Devices, Research Division, Bonn, Germany; 2 University of Bonn, University Hospital Bonn, Institute for Medical Biometry, Informatics and Epidemiology, Bonn, Germany; 3 Federal Institute for Drugs and Medical Devices, Pharmacovigilance Division, Bonn, Germany; 4 Department for Dermatology and Allergy, University Hospital RWTH Aachen, Aachen, Germany; University of Health and Allied Sciences, GHANA

## Abstract

**Introduction:**

The vigiGrade completeness score established in VigiBase, the worldwide ADR database, measures the completeness of information provided in the structured format of each ADR report. Currently, no such measurement is implemented in the European ADR database EudraVigilance. The aim of our study was to adjust the algorithm of the vigiGrade completeness score to the structure of the ADR reports from EudraVigilance (=Vigi4Eudra-score).

**Materials and methods:**

An R script was developed to semi-automatically assess the completeness of structured information provided in the ADR reports extracted from EudraVigilance. The script was applied to four different datasets. We compared the values achieved by the Vigi4Eudra-score to those of the vigiGrade completeness score per ADR report.

**Results:**

Approximately 15,000 spontaneous reports from the four datasets were assessed. The mean values of the Vigi4Eudra-score of all four datasets ranged from 0.41–0.59. Compared to the values of the vigiGrade completeness score, most commonly, none or only minor discrepancies were found. These were largely attributed to the categories that needed to be adapted for use within EudraVigilance.

**Conclusions:**

We provide an R script to assess the completeness of information provided in the structured format of ADR reports from EudraVigilance, which may be useful for researchers, pharmaceutical companies, and regulatory authorities. Our use cases demonstrate its successful application.

## 1 Introduction

Spontaneous adverse drug reaction (ADR) reports are an important source of information for the benefit-risk assessment of medicinal products in pharmacovigilance practice. These are stored in ADR databases where disproportionality analyses are applied to identify safety signals. Safety signals can arise, for example, when a new ADR occurs or when new aspects of a known ADR become apparent including, among others, changes in the frequency, distribution (e.g., sex, age and country), duration, severity or outcome of the ADR. These signals do not imply causality. Thus, an individual case assessment of the ADR reports is needed as part of the signal validation and assessment procedure. If the signal is valid, it will be assessed and, for example, may trigger further studies, lead to amendments of the product information, or even result in the withdrawal of the medicinal product from the market [1–5].

Given the considerable increase in the number of spontaneous ADR reports over the past years, the individual assessment of these reports is becoming increasingly challenging. Approaches involving (partially) machine-based evaluations of these reports, or prioritization of particularly valuable reports, may therefore be helpful [6,7].

As an example, the vigiGrade completeness score established by the Uppsala Monitoring Centre is one tool to measure the completeness of information provided [8]. It was developed for the assessment of ADR reports in VigiBase, the World Health Organisations global database of individual case safety reports [9]. VigiBase contains ADR reports from all over the world.

A study of our research group demonstrated that ADR reports assessed as having an at least possible causal relationship exhibited higher values of the completeness score than those for which the causal relationship was deemed as “not assessable” [10]. Hence, a quantification of the information included could serve as a basis for the prioritisation regarding the sequence of reports to be evaluated by the assessors [11,12].

The first aim of our study was to create a score (Vigi4Eudra-score) based on the algorithm of the vigiGrade completeness score adjusted to meet the structure of ADR reports from EudraVigilance. Based thereon, we created an R script/application to evaluate the completeness of large datasets of ADR reports from EudraVigilance with a small number of interventions by the user. Our second aim was to demonstrate the feasibility of the script/application based on four exemplarily chosen datasets, each having a different focus regarding the reports included. As a third aim, we investigated whether the values of the Vigi4Eudra-score differed from those of the original vigiGrade completeness score for each ADR report.

In this manuscript, we introduce the script/application and make it publicly available. All users with appropriate access to EudraVigilance like researchers, competent authorities and pharmaceutical companies, could potentially benefit from this approach and its application.

## 2 Materials and methods

### 2.1 EudraVigilance

ADRs are noxious and unintended responses to medicinal products occurring within or outside the terms of the marketing authorization (e.g., off label use) [13]. ADRs can be reported by healthcare professionals (HCP) (e.g., pharmacists, physicians, nursing staff) as well as non-healthcare professionals (non-HCP) (e.g., consumers, lawyers) [14,15]. In contrast to clinical trials, where drug administration and ADR recording follow a predefined protocol, spontaneous reports are submitted voluntarily from everyday clinical practice. Further information regarding the reporting channels of these ADR reports can be found elsewhere [13,16].

All spontaneously submitted ADR reports from the member states of the European Economic Area (EEA) are included in the ADR database EudraVigilance, which is operated by the European Medicines Agency (EMA) [13]. These spontaneous ADR reports have to include at least an identifiable reporter and patient, one suspected drug and one suspected ADR [13]. EudraVigilance contains also other types of ADR reports (e.g., from clinical trials) that are not the subject of our study. Notably, different levels of access to EudraVigilance are provided to different stakeholders [17].

#### 2.1.1 EudraVigilance data analysis system (EVDAS).

ADRs reports can be extracted from EudraVigilance in a line listing [18–21]. This is a tabular compilation of all ADR reports included in the respective dataset. Each individual report is listed in one row, and each column contains a specific category of information, for example, the date of receipt. If there is no information available for a certain category in the report, a placeholder like “not available”/”not specified” is used to designate its absence. The line listing can be exported in the format of.csv or.xlsx. In this manuscript, we cannot provide an exemplary line listing due to data privacy requirements.

Each ADR report can consist of structured and unstructured free text information (e.g., narratives). The latter can include additional information, e.g., a more detailed description of the ADR. In the line listing only the first 3000 characters of these narratives are included.

### 2.2 VigiBase and the vigiGrade completeness score

The vigiGrade completeness score is a tool to measure the presence of selected clinically relevant information provided in the structured format of ADR reports in VigiBase [8,9]. From this database line listings can be extracted, too, which include the calculated vigiGrade completeness score for each ADR report [8].

The vigiGrade completeness score comprises ten categories of information, which are assigned to three categories of importance. Each report is reviewed to determine the presence or absence of information for these variables. Reports without any information assigned to a specific category are penalised with the following values, reflecting the assigned degree of importance of each category (Table 1):

**Table 1:** Comparison of penalties of the original vigiGrade completeness score and penalties of the automated Vigi4Eudra-score for line listings from EudraVigilance.

Information	Description	Penalty (options) according to vigiGrade completeness score penalty [8]	Penalty according to our Vigi4Eudra-score (automated script)	Basis for decision of our R script
Time to onset	The time that elapses between the intake of the suspected drug and the occurrence of the ADR	50% imprecise or no information 30% uncertainty exceeds 1 month 10% otherwise	50%	For each ADR-drug combination, the time to onset based on the start date of the drug and the reaction date of the ADR is calculated. If the time to onset of ≥0 days cannot be calculated, it is considered unavailable and a penalty of 50% is applied. No further distinctions of uncertainties can be made.
Indication	The indication of the suspected drug	30%	30%	If unknown or unavailable, the penalty will be deducted.
Outcome	Description of the consequences of the reaction	30%	30%	If unknown or unavailable, the penalty will be deducted.
Sex	The sex of the patient	30%	30%	If unknown or unavailable, the penalty will be deducted.
Age	The age of the patient	30% no information 10% if only age group is specified	30%	If unknown or not available, the 30% penalty is deducted. Please note that no age groups are given in the structured format of the EudraVigilance line listing.
Dose	The dose of the suspected drug	10%	10%	If unknown or not available, the penalty is deducted. All other numeric values, regardless of their unit (e.g., mg, units, ml, tablets) are counted as available.
Country	The country of origin of the person who created the report	10%	10%	If unknown or unavailable, the penalty will be deducted.
Primary reporter	Qualification of the person(s) who reported the ADR (e.g., physician, pharmacist)	10%	10%	If unknown or unavailable, the penalty will be deducted.
Report type	Indicates the type of the report (spontaneous report, report from study, other)	10%	10%	If unknown or unavailable, the penalty will be deducted.
Comments	Also called narrative. Contains unstructured free text information	10%	10%	If the narrative including the reporter’s comment, and sender’s comment is not available, the penalty is deducted.

- 50% (time to onset)
- 30% (indication of the drug therapy, ADR outcome, sex and age of the patient)
- 10% (country of origin, qualification of the person who created the report, report type, narrative).

Notably, the vigiGrade completeness score is calculated for each ADR-drug combination of a report by multiplying the fixed “start value” = 1.0 with all penalties of missing information (e.g., for missing sex = 30%, multiplication by 0.7. For all penalty values please refer to Table 1):


$$\begin{array}{c} vigiGrade\;completeness\;score=\prod {\mathrm{\backslash nolimits}}_{i=\text{1}}^{\text{1}\text{0}}\left(\text{1}-{Penalty}_{i}\right)=\left(\text{1}-P_{\text{1}}\right)\ldots \left(\text{1}-P_{\text{1}\text{0}}\right) \end{array}$$

To determine the overall value of the vigiGrade completeness score for each report, the mean of all values for each ADR-drug combination within the report is calculated. Importantly, the mean is only calculated for suspected and interacting but not for concomitant drugs.

The final value ranges from 0.07 (no information evaluated by the vigiGrade completeness score included) to 1.0 (all that information included). Reports with a value equal to or greater than 0.8 are considered well-documented by the authors [8].

### 2.3 Automated application of the vigiGrade completeness score adapted to line listings from EudraVigilance

An R script [22] was written to automatically evaluate the completeness of structured information provided in spontaneous ADR reports related to drugs (vaccinations and hyposensitisation solutions are excluded) extracted as line listing from EudraVigilance. To this purpose, the algorithm of the vigiGrade completeness score was used as a basis.

Two (minor) adaptations to the penalties of the vigiGrade completeness score to meet the format of the line listing extracted from EudraVigilance were performed. First, information on the age group of the patient is shown in the line listing from VigiBase but not in that of EudraVigilance. Thus, in EudraVigilance a distinction is merely made whether an exact indication of age is present or not. Second, the time to onset is not explicitly included as such in the line listing extracted from EudraVigilance, but it can be calculated if the dates regarding the start of drug therapy and the occurrence of the ADR are available in the format DD/MM/YYYY. Consequently, the time to onset can only be calculated precisely or not at all (i.e., a binary treatment), since inaccurate values (e.g., MM/YYYY, or YYYY) are not provided in the line listing. The same applies to the implemented time to onset matrix in the individual case safety report (ICSR) form. For its generation start dates of drug(s) and reaction(s) also have to be available in the valid format DD/MM/YYYY. Thus, this limitation is not associated with the line listing itself but results from the absence of the necessary data in the valid format. Thus, the “lenient” penalties for these two categories as utilised in the original vigiGrade completeness score cannot be applied to the line listing from EudraVigilance. In summary, the binary treatment of the variable time to onset reflects inherent limitations of the structured information provided in EudraVigilance which extends to the line listing.

The application can be used for all line listings exported from EudraVigilance in the format of.xlsx or.csv. In the following, the resulting score is referred to as Vigi4Eudra-score, an abbreviation for “*vigiGrade completeness score adapted for the assessment of ADR reports from EudraVigilance*”.

#### 2.3.1 R and RStudio.

The application for automatically evaluating the completeness of each ADR report was created and is implemented using RStudio, an integrated development environment based on the programming language R [22,23]. R and RStudio are available as open-source versions and can be downloaded free of charge for Windows, Mac and Linux (https://posit.co/download/rstudio-desktop/). Predefined functions are available by activating so-called add-on packages.

For our R script the following add-on packages are used:

- **dplyr** [24]
- **tidyr** [25]
- **stringr** [26]
- **lubridate** [27]
- **purrr** [28]
- **readxl** (Only for Excel (.xlsx) files) [29]

#### 2.3.2 Description of our application.

The R script, a description of the required steps, and an overview of the automatically generated export-files can be found in the R files and S1 Text of this manuscript. The application follows these five steps:

1. All reports referring to vaccines and hyposensitisation solutions are removed, since we wanted to focus solely on drugs.
2. A separate row for each ADR-drug combination of each ADR report in the dataset is created, listing all information evaluated in the original vigiGrade completeness score.
3. The presence/absence of certain information is assessed according to Table 1.
4. Analogous to the vigiGrade completeness score, the mean value of the completeness score for each ADR report is calculated based on all values for each ADR-drug combination contained in this report.
5. Various documents are automatically created containing detailed results for each ADR report, and each ADR-drug combination (S1 Text).

Table 1 is based on the original vigiGrade completeness score publication (vigiGrade: A Tool to Identify Well-Documented Individual Case Reports and Highlight Systematic Data Quality Issues by Bergvall et al. [8] – which is distributed under the terms of the Creative Commons Attribution-NonCommercial 4.0 International License) and modified to allow a better comparison of similarities and differences of both scores.

### 2.4 Datasets used to demonstrate our application of the Vigi4Eudra-score

We tested the Vigi4Eudra-score in four exemplary datasets related to different patient populations or specific ADRs.

#### 2.4.1 Selection of datasets.

**Fig 1:**
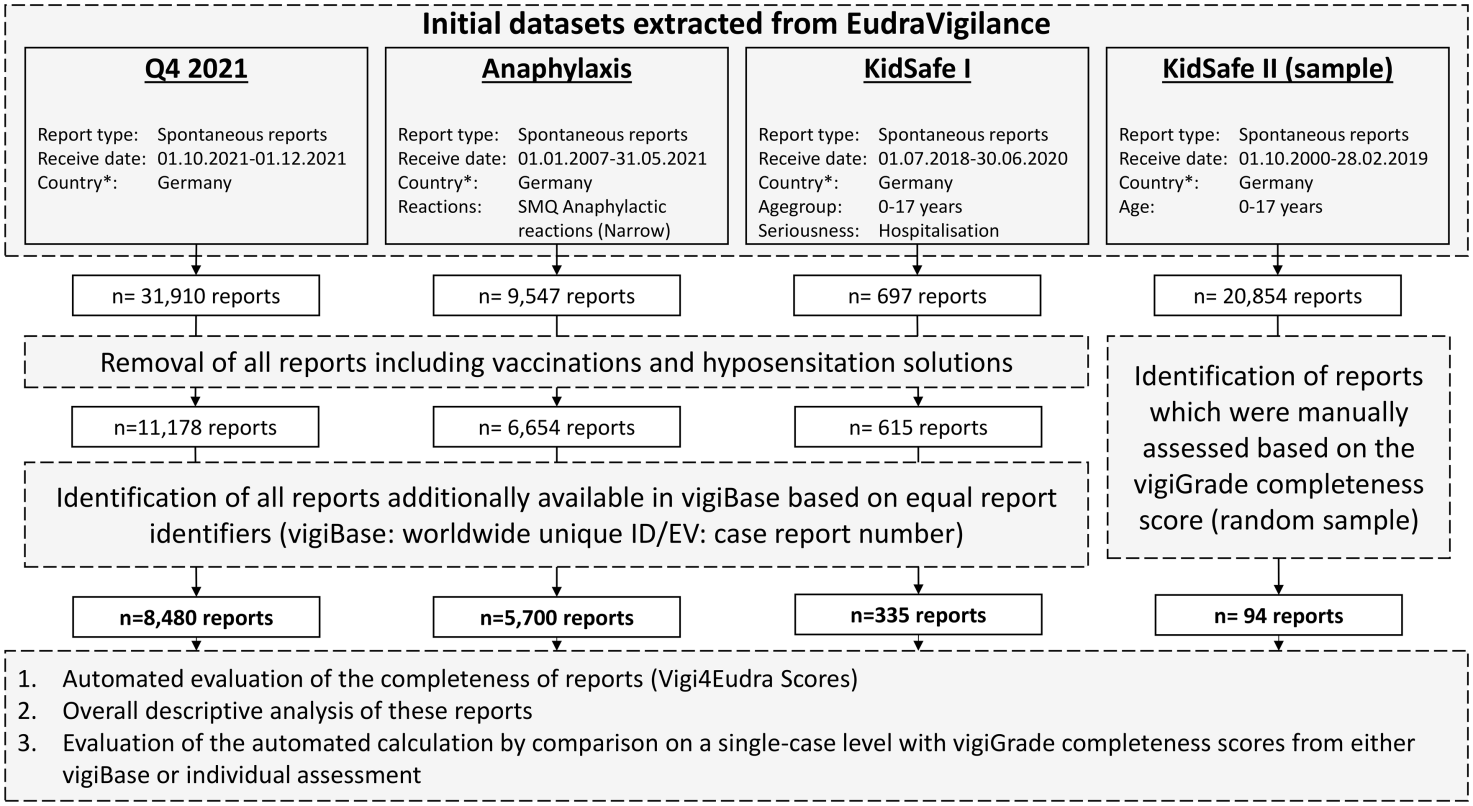
Flowchart: identification of reports for all four datasets. Legend: n = number of reports; SMQ: Standardized MedDRA Queries are pre-defined collections of MedDRA codes (in this case potential ADRs) corresponding to a specific diagnosis or condition (in this case Anaphylaxis) [34].

We evaluated the Vigi4Eudra-score using four datasets of previous projects of our working group [30–33]. Further descriptions of these datasets can be found in the relevant literature. In brevity, those four datasets comprised: a) all spontaneous ADR reports from Germany received during the 4^th^ quarter of 2021 (designated as: Q4 2021) b) all spontaneous ADR reports of anaphylactic reactions received between 2007 and 2021 (designated as: Anaphylaxis) c) spontaneous ADR reports exclusively involving children received between 07.2018 and 06.2020 (designated as: KiDSafe I) and, d) a sample of spontaneous ADR reports referring to children with hospitalisation or prolongation thereof received between 2000 and 2019 (designated as: KiDSafe II) (Fig 1). Please note that, aside from nine reports included in both the Anaphylaxis and KiDSafe I datasets, no other reports were found in more than one of the four datasets.

We have selected these datasets as they all represent different but likely datasets used for analyses of spontaneous reports. Dataset Q4 2021 comprises a broad, large dataset providing a snapshot of the database at a particular timepoint. In contrast, the other datasets include prototypes of a rare and relevant ADR (Anaphylaxis) or a restricted target population (KiDSafe I). KiDSafe II differs from the other datasets in that all reports were individually assessed, and it thus serves to evaluate the differences between individual assessments and automated evaluations. (see section 2.6). It is derived from a descriptive analysis of ADR reports in children and adolescents from Germany, which included about 21,000 spontaneous ADR reports retrieved from 01. January 2000–28. February 2019 [33]. Out of these, a random sample of 100 ADR reports (0.5% (100/20,854)) was assessed regarding their quality (=completeness) and the causal association between the reported ADRs and the applied drugs. This random sample was used in the present study.

For all datasets, only those reports identified in VigiBase (see below) or assessed during the individual assessment were included in the final datasets and analysed accordingly.

#### 2.4.2 Identification of reports in VigiBase.

All report identifiers (EudraVigilance: *case report numbers)* from the datasets Q4 2021, Anaphylaxis and KiDSafe I (excluding vaccines and hyposensitisation solutions) were extracted. We searched for corresponding reports in VigiBase based on these report identifiers (ID) (VigiBase: worldwide unique ID) and subsequently extracted matching reports. As mentioned in section 2.2, a vigiGrade completeness score is assigned to each report in VigiBase. Ultimately, 8,480/11,178 reports (75.9%) of the Q4 2021, 5,700/6,654 (~85.7%) reports of the Anaphylaxis and 335/615 (~54.5%) reports of the KiDSafe I dataset were found and extracted from VigiBase.

Regarding the individually assessed KiDSafe II dataset, 94.0% of the reports were matched, based on equal case report numbers (n = 94 reports). Notably, during the individual assessment of spontaneous reports within the KiDSafe project, only the leading ADR-drug combination of each report was considered when calculating the completeness score [33]. Other ADR-drug combinations, if reported, were not considered, as they either had a less likely causal association or were co-reported with the leading ADR. Contrary to that, the automated assessment of the Vigi4Eudra-score generates a mean value of all individual values for each ADR-drug combination.

### 2.5 Application and comparison of scores

The automated application of the Vigi4Eudra-score was applied to all four datasets described above. Mean values including standard deviations (SD) and median values with their interquartile ranges (IQR) were calculated for each dataset. Furthermore, the ranges of the values of the Vigi4Eudra-score, the number of different values of the Vigi4Eudra-score for each ADR-report and the number of ADR reports with a value of the Vigi4Eudra-score ≥0.8 were determined.

### 2.6 Validation of Vigi4Eudra-score

External validation was performed for the datasets Q4 2021, Anaphylaxis and KiDSafe I by comparison of the values of the Vigi4Eudra-score to those of the vigiGrade completeness score deposited in ADR reports from VigiBase (reference). The internal validation of the KiDSafe II dataset was based on the comparison of the values of the automated Vigi4Eudra-score and the vigiGrade completeness score rated by individual single report assessments of our working group.

For both comparisons, the differences between the automatically assigned values of the Vigi4Eudra-score and the values of the reference dataset were calculated. The average differences were characterised by calculating their mean and median values with their SD and IQR.

As indicators for the conformity of the values of the Vigi4Eudra-score (EudraVigilance reports) and the reference values, interclass correlation coefficient (ICCs) with 95% confidence intervals (CI) were calculated [35].

### 2.7 Evaluation of deviations in the external validation

All reports with a deviation of ≥ + 0.3 or ≤ −0.3 between the values of the Vigi4Eudra-score (EudraVigilance reports) and the vigiGrade completeness score (VigiBase reports) were further investigated (Q1 2021: n = 446, 5.3%; Anaphylaxis: n = 408, 7.2%; KiDSafe I: n = 18, 5.4%). To this purpose, the line listings of these reports from EudraVigilance to those extracted from VigiBase were compared. We specifically aimed to determine whether the differences arose from adjustments to our application (age groups, time to onset).

For those reports in which no differences were found on this superior level of the ADR report further analyses on the ADR-drug combination level were performed. Notably, differences may be related to the categories of dose, indication, outcome or time to onset since these can be specified individually for each drug and each ADR. Note that the presence of free text information could not be considered within this evaluation as free text descriptions are not included in the line listings extracted from VigiBase.

### 2.8 Comparison of the best value of the Vigi4Eudra-score per report with individual evaluation

As described in 2.4.2 only the leading ADR-drug combination was evaluated for each report in the individual assessment of the KiDSafe II reports. We assume that the leading ADR-drug combination may be the one documented most completely (especially in terms of time to onset, drug indication, etc.). Thus, we compared the value of the score from the individual assessment to the average value of the Vigi4Eudra-score (see section 2.6) and to the highest automatically generated value of the Vigi4Eudra-score (best score). The same calculations were performed as described in section 2.6.

## 3 Results

We assessed the values of the Vigi4Eudra-score of 8,840 reports in the Q4.2021 dataset, 5,700 reports in the Anaphylaxis dataset, 335 reports in the KiDSafe I dataset, and 94 reports in the KiDSafe II dataset.

### 3.1 Assessment of values calculated by the automated Vigi4Eudra-score

**Fig 2:**
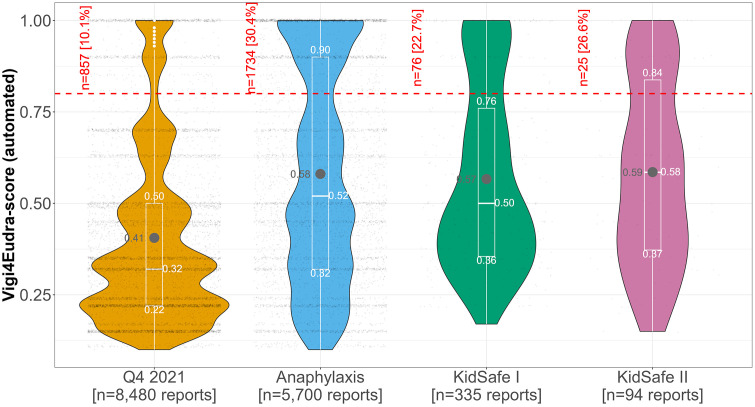
Violin plot and box plot of all key figures for the values of the Vigi4Eudra-score. Legend: Violin plot showing the distribution of the values of the Vigi4Eudra-score for each dataset. Grey dots and numbers indicate the mean value. White numbers indicate key values of the 25% quantile, median, and 75% quantile displayed by the box plot. Small black dots indicate the value(s) of each report. White dots indicate outliers. The red dotted line shows the threshold of well-documented reports ≥0.8. The numbers and percentual shares for all reports depicted above the threshold fulfilling the well-documented criterion of each dataset are indicated in red.

Except for the Q4 2021 dataset (mean = 0.41; SD ± 0.24), the mean values of all Vigi4Eudra-score were approximately 0.6 in all datasets (SD: Anaphylaxis ±0.29; KiDSafe I ± 0.25; KiDSafe II ± 0.25) (Table 2, Fig 2). In the datasets Q4 2021 and Anaphylaxis, the values ranged from 0.10 to 1.00. For both KiDSafe datasets it ranged from 0.15 and 0.17 to 1.0. However, the distribution of the values differed (see violin plot in Fig 2).

**Table 2:** Distribution of values of the Vigi4Eudra-score in the four exemplary datasets.

	Q42021 [n=8480]	Anaphylaxis [n=5700]	KiDSafe I [n=335]	KiDSafe II [n=94]
Statistical key findings of the values of the automated Vigi4Eudra-score				
**Mean (SD)**	0.41 [±0.24]	0.58 [±0.29]	0.57 [±0.25]	0.59 [±0.25]
**Median [IQR]**	0.32 [0.22 - 0.50]	0.52 [0.32 - 0.90]	0.50 [0.36 - 0.76]	0.59 [0.37 - 0.84]
**Range**	0.10 - 1	0.10 - 1	0.17 - 1	0.15 - 1
Well-documented [values of the Vigi4Eudra-score ≥0.8]				
**Number of reports [percentage]**	857 [10.1%]	1734 [30.4%]	76 [22.7%]	25 [26.6%]
Different values of the Vigi4Eudra-score for ADR-drug combinations within a single report				
**Number of different values of the Vigi4Eudra-score per report**	**Number and percentual share of reports in each dataset including the respective number of different values of the Vigi4Eudra-score per report**			
**One value calculated or no difference between the values** ^**a**^	6656 [78.5%]	3867 [67.8%]	175 [52.2%]	63 [67.0%]
**2**	1476 [17.4%]	1280 [22.5%]	109 [32.5%]	26 [27.7%]
**3**	189 [2.2%]	256 [4.5%]	25 [7.5%]	5 [5.3%]
**4**	117 [1.4%]	190 [3.3%]	13 [3.9%]	–
**≥5**	42 [0.5%]	107 [1.9%]	13 [3.9%]	–

SD = standard deviation, IQR = interquartile range.

^a^ Either only one value of the Vigi4Eudra-score could be calculated, e.g., in case of only one ADR-drug combination reported or all ADR-drug combinations reported received the same value of the Vigi4Eudra-score.

The highest proportion of well-documented reports (value of the Vigi4Eudra-score ≥0.8) was observed in the Anaphylaxis (n = 1,734; 30.4%) and the lowest in the Q4 2021 dataset (n = 857; 10.1%).

In approximately four out of five reports in the dataset Q4 2021 (n = 6,656; 78.5%), only one value of the Vigi4Eudra-score was calculated, either because there was only one ADR-drug combination or because multiple ADR-drug combinations were present, but all yielded the same value. In contrast, this only applied to about half the reports in the KiDSafe I dataset (n = 175; 52.2%).

### 3.2 Comparison of automatically calculated values of the Vigi4Eudra-score with those of the vigiGrade completeness score

**Fig 3:**
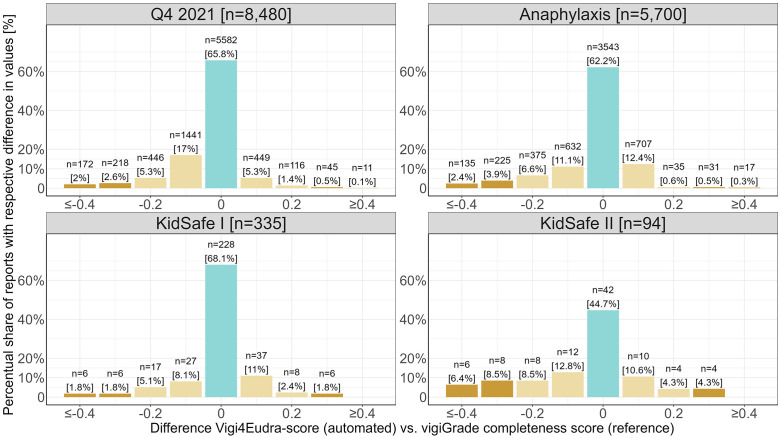
Differences between the automatically calculated values of the Vigi4Eudra-score and the values of the respective reference. Legend Fig 3: Bar chart showing the distribution of the differences between the values of the Vigi4Eudra-score and the vigiGrade completeness score. Above each bar, the total number of reports included and its percentual share within the respective dataset is indicated.

The median difference between the values of the automated Vigi4Eudra-score compared to the (internal or external) reference values was 0 in all datasets (IQR: Q4 2021–0.06 to 0; Anaphylaxis −0.05 to 0; KiDSafe I 0 to +0.02; KiDSafe II −0.13 to +0.02) (S1 Table, Fig 3). With −0.01 [±0.11], the lowest mean difference was found for the KiDSafe I dataset, while the highest difference (−0.05 [±0.17]) was found for the KiDSafe II dataset (individually assessed reports).

Approximately only one in twenty reports from the datasets compared to an external reference showed a deviation of ≥ + 0.3 respectively ≤ −0.3 (Q4 2021: n = 446, 5.3%; Anaphylaxis: n = 408, 7.2%; KiDSafe I: n = 18, 5.4%). In contrast, this was the case in approximately one in five reports of the KiDSafe II dataset (0 = 18; 19.1%) (see discussion).

Intraclass Correlation Coefficients (ICC) of ≥ 0.90 (Q4 2021, KiDSafe I, Anaphylaxis) were calculated for those datasets with external reference. Notably, the ICC for the KiDSafe II dataset was smaller (0.79 [0.65–0.84].

### 3.3 Exploration of larger differences in the external validation

Among those reports with differences of ≥ + 0.3 or ≤ −0.3 (S3 Table), the deviation ≤ −0.3 (Q4 2021: n = 390/446, 87.4%; Anaphylaxis: n = 360/408, 88.2%; KiDSafe I: n = 12/18, 66.7%) was more frequent than the deviation ≥ +0.3.

In 100.0% of the KiDSafe II (n = 18/18), 89.0% of the Anaphylaxis (n = 363/408) and 85.9% of the Q4 2021 (n = 383/446, 85.9%) dataset, differences were found on the report level (S1 Fig, S3 Table).

Especially discrepancies regarding the time to onset (Q4 2021: n = 182/446, 40.8%; Anaphylaxis: n = 99/408, 24.3%; KiDSafe I: n = 5/18, 27.8%) and age (Q4 2021: n = 112/446, 25.1%; Anaphylaxis: n = 80/408, 19.6%; KiDSafe I: n = 6/18, 33.3%) were identified on the report level as the main reasons (S1 Fig, S3 Table).

In the remaining reports, differences on the ADR-drug level were found in 9.2% of the Q4 2021(41/446), in 36.0% of the Anaphylaxis (147/408) and 22.2% of the KiDSafe I (4/18) dataset. Discrepancies on the ADR-drug level were mainly based on the time to onset (Q4 2021: n = 24/41, 58.5%; Anaphylaxis: n = 138/147, 93.9%; KiDSafe I: n = 3/4, 75%) (S4 Table). A detailed evaluation is shown in S2–S4 Tables and S1 Fig.

### 3.4 Comparison best value of the Vigi4Eudra-score per report vs. individual evaluation (KiDSafe II)

Compared to the values of the *best* Vigi4Eudra-score automatically assessed for each report, the differences to the individual assessment in the KiDSafe II dataset ranged from −0.41 to 0.37 with a mean difference of −0.01 (SD ± 0.16) (S5 Table). The *best* value of the Vigi4Eudra-score indicates a marginally stronger concordance along with more reports showing an exact agreement (n = 54, 57.4% vs. Vigi4Eudra-score mean: n = 42, 44.7%) compared to using the mean value of the Vigi4Eudra-score (ICC (0.82 versus 0.76) (see section 3.2).

## 4 Discussion

We developed an R script that enables the automated assessment of the completeness of ADR reports exported as a line listing from EudraVigilance. This script/application is based on the principles of the vigiGrade completeness score established in VigiBase. Consequently, the penalties for the categories age groups and time to onset were adjusted, because these data are not uniformly reported in the line listings of ADR reports from EudraVigilance and VigiBase. The application provided here was already used by our working group in several other analyses [10,30–33].

The R script can be found in the attached files that have to be implemented in R and saved in the same folder as the datasets as described (S1-S8 Files). Our aim was to share the principles of this approach with other researchers, competent authorities or pharmaceutical companies having appropriate access to line listings extracted from EudraVigilance. It can be used to examine large datasets requiring only a few steps carried out by the user.

### 4.1 Validation of the Vigi4Eudra-score

In summary, only marginal deviations were found in our use cases (S1 Table) when comparing the values of the Vigi4Eudra-score to those of the vigiGrade completeness score or the individual assessment confirming the feasibility of our automated approach.

The high degree of agreement between the values of the Vigi4Eudra-score and the vigiGrade completeness score was also confirmed by the calculated ICC values which can be interpreted as good (Q4 2021 and KiDSafe I) to excellent (Anaphylaxis) [35].

Discrepancies between both scores were merely identified in a small proportion of reports for each dataset. These discrepancies were primarily attributed to differences in how the two algorithms evaluated and scored age and time to onset. Another possible reason could be differences in the availability of information between the two databases, EudraVigilance and VigiBase [36].

The slightly lower level of agreement between the values of the individual assessment and the values of the Vigi4Eudra-score for the KiDSafe II dataset, is presumably related to the fact that information provided in the free text (narrative) of the reports was considered in the individual assessment, too. In addition, the individual assessment focused solely on the evaluation of the leading ADR-drug combination and did not involve all ADR-drug combinations. We tried to address this issue by comparing the value of the best score with the leading ADR. Here, the differences were apparently smaller, and concordance increased regarding the ICC value, which was assessed as *good* in both analyses [35]. Hence, this comparison delivered some re-assurance although the comparison is still not adequately head-to-head. Notably, it is possible that the “leading” ADR-drug combination of a report might not be the one with the highest value (as we assumed). Further studies are needed to investigate if the differences between the values for different ADR-drug combinations per ADR report are related to differences between the information given for the “leading ADR-drug combination” and other co-reported ADR-drug combinations.

### 4.2 Advantages of the Vigi4Eudra-score

Our application itself is structured in a way to reduce the workload for assessors (see “instruction of the application” in the S1 Text). Except for the nine steps to “activate” the application, the assessment itself is carried out automatically without any further assistance from the user.

A major advantage of our application lies in its 100% reproducibility when repeatedly applied to the same dataset compared to an individual assessment, which is likely to be more prone to errors. Furthermore, the evaluation of the datasets can be completed in a very short time, e.g., around 90 seconds for 30,000 reports tested on several “everyday life” notebooks/computers. However, the time required strongly depends on the size of the dataset, and the computer used.

Our automated assessment not only reveals the final value of the score of each ADR report, but also the individual values for all ADR-drug combinations within a report. This might be of importance for specific pharmacovigilance questions. Additionally, the document “*FullCalculation_PerReactionDrug.xlsx*” allows an easy single-report specific review, e.g., by using the Excel filter function for the presence or absence of specific information (such as age, sex, or outcome), which could be crucial for further evaluation.

The R script was created for the evaluation of ADR reports from EudraVigilance. However, the script could potentially be used for the assessment of the completeness of structured information provided in ADR reports from other databases if the respective categories align. Probably adjustments to column names and operators will be necessary.

The application itself does not require access to the internet, except for the one-time download of the software R, RStudio, the required packages, and the line listing [37,38].

The provided R code is in principle adaptable to future changes of the EudraVigilance line listing. In addition, content-related changes would not come abruptly but with appropriate lead time which would facilitate respective amendments. Finally, we cannot guarantee the long-term maintenance of the R script, as this also depends on funding which cannot be foreseen.

### 4.3 Limitations of the Vigi4Eudra-score/Application

First, the access to the line listing from EudraVigilance differs for different stakeholders [17]. Currently, our application cannot be used for superordinate line listings extracted via public access [19].

Furthermore, our application and validation have been carried out for the assessment of spontaneous reports only, excluding those related to vaccines and hyposensitization solutions. Although several experimental test-runs have shown that the assessment of other reports (e.g., from clinical trials) and spontaneous reports containing vaccines or hyposensitisation solutions (by deletion of “source code 3” lines 69–116) was also feasible, the lack of validation precludes any statements on their accuracy. Further validations are needed to assess the feasibility.

The application can be applied to the current format of line listings (March 2024) extracted from EudraVigilance. However, changes in the structure, e.g., by renaming columns or changing separators between varying information, could lead to errors. In this case, the code needs to be adapted. The R script was written in way, that the application should be capable of dealing with minor changes if specific key words remain in the title of the columns.

Finally, the Vigi4Eudra-score only considers if free text information was provided but not the content reported. However, neither does the vigiGrade completeness score. Since it was the aim of our study to create a score (Vigi4Eudra-score) based on the algorithm of the vigiGrade completeness score, we do not consider this as a limitation. However, both approaches could benefit from advanced methods, such as artificial intelligence, that systematically extract and analyse relevant information from the narrative. This information could then – if missing – be added to the structured fields which are considered by both scores.

## 5 Conclusions

Based on the analyses presented in this manuscript, we were able to demonstrate the feasibility of our application for assessing large datasets of spontaneous reports from EudraVigilance. This application enables researchers, pharmacovigilance experts and others to gain an overview of their dataset in the shortest possible time regarding the a) overall presence of selected, clinically relevant information within the whole dataset, b) within each single report, c) on the level of ADR-drug combinations and d) differences of values within each single report (mean value vs. individual values for each ADR-drug combination). The approach could be used to prioritise ADR reports for signal validation purposes as reports with an al least possible causal relationship may obtain a higher value of the Vigi4Eudra-score as seen in another study [10].

## Supporting information

S1 Text Instruction of the application. (DOCX)

S1 Table Detailed analysis of the distribution of the values of the Vigi4Eudra-score in the four exemplary datasets. (DOCX)

S2 Table Analyses of reports with differences of ≥ + 0.3 or ≤ −0.3 between the values of the Vigi4Eudra-score and the vigiGrade completeness score. (DOCX)

S3 Table Discrepancies on the ADR report level. (DOCX)

S4 Table Discrepancies on the ADR-drug level. (DOCX)

S5 Table Comparison mean value and best value of the Vigi4Eudra-score to the individual assessment. (DOCX)

S1 Fig Differences identified in the information provided per category. (DOCX)

S1 File User File Vigi4EudraScore_xlsx. (DOCX)

S2 File Source1_.xlsx. (DOCX)

S3 File Source2_.xlsx. (DOCX)

S4 File Source3_.xlsx. (DOCX)

S5 File UserFile_Vigi4EudraScore. (DOCX)

S6 File Source1_.csv. (ZIP)

S7 File Source2_.csv. (DOCX)

S8 File Source3_.csv. (ZIP)

## Abbreviations

ADR: adverse drug reaction
EEA: European Economic Area
EMA: European Medicines Agency
EV: EudraVigilance
EVDAS: EudraVigilance data analysis system
HCP: healthcare professionals
ICC: Intraclass Correlation Coefficient
ID: identifier
IQR: interquartile range
Non-HCP: non-healthcare professionals
SD: standard deviation
SMQ: Standardized MedDRA Queries
WHO: World Health Organisation
