# Supplementary material for: Vigi4Eudra-score: Evaluation of the completeness of spontaneous adverse drug reaction reports in EudraVigilance
Source: PLoS One. 2026 Feb 25;21(2):e0343694. doi: 10.1371/journal.pone.0343694 (PMC12935194; doi:10.1371/journal.pone.0343694)
Supplement: S1 Text — (DOCX) [file pone.0343694.s001.docx]

# **S1 Text. Instruction of the application.**

Before the application made available by us can be used the user must download the programmes R and R-Studio, which can be done e.g. here: <https://posit.co/download/rstudio-desktop/>.

After the first installation of R and R-Studio the application can be run by the user by just opening the user file and carrying out nine relevant steps described in the following. The code relies on three source code files, which do not require to be opened themselves. If they are stored one folder, they are automatically activated by the *source* functions in the user file and carry out the automated application of the Vigi4Eudra-score on their own.

Prior the explanation of each step, a brief explanation on how each of the code/application components is activated is provided. Individual steps are activated by marking the required line of code (triple-click with the left mouse button, or hover over it with the left mouse button hold down) and subsequently selecting the "run" button in the top right-hand corner of the script. Another option is to activate selected lines using the shortcut Ctrl+ Enter.

##### **1^st^ step**

The user should place the line listing they want to analyse in a folder together with the user file and all three source codes. It is recommended to generate a new folder for each application on a new line listing.

##### **2nd step**

Now the user file can be opened in R-Studio.

##### **3^rd^ step**

The first command *rm(list=ls())* (line 16) has to be activated to remove any existing values from previous analyses in R from the cache (R-Studio environment).

##### **4^th^ step**

The user has to specify the file path of the folder containing a) the user file, b) the three source codes and c) the line listing, which shall be processed within the inverted commas of the *setwd("B:/Example/Example/Example/Example")* command. Please ensure that all slashes included in the file path are forward slashes “**/**” instead of backslashes “**\**”. Subsequently the user must activate this command.

##### **5^th^ step**

The user has to mark and execute the *source ("Source1_csv.R")* command. As soon as the command has been successfully executed the information *"Excellent, all the source codes required are available in your specified file path. All packages required for the further processing were installed and should be activated by now. You can go on!"* appears in the R-Studio console (usually at the bottom left in R-Studio).

##### **6^th^ step**

The user has to enter the name of the line listing file between the quotation marks in *Dataframe1<-read.csv2 ("Example.csv",sep=",")*. Please ensure that the (correct) file format .csv or .xlsx is indicated in the file name, otherwise the code will not work. Once you have entered the file name, you can also execute this command. This will read the line listing file into R-Studio.

##### **7^th^ step**

The *source ("Source2_csv.R")* command can be marked and executed. Once this command has been executed successfully, the console displays the message *"In process control successful. The application was able to identify the right number of columns."&"You can go on with step 3!"*.

##### **8^th^ step**

Finally, the user can insert a name for the dataset being evaluated between the inverted commas in the *Dataframename<-paste("Designated_Name_For_Resultsfiles")* command, where the placeholder "*Designated_Name_For_Resultsfiles*" appears. This name will be added to all automatically created documents generated by the application. Please make sure to use a short but unique name and avoid the use of special characters, as this can lead to the abortion of further processing! Recommendation: Ideally, you should use unique designations comprising, for example, the date as a numerical sequence and a brief description of the content, e.g. "20231224AllergicReactions". If necessary, underscores "**_**" are recommended as separators.

##### **9^th^ step**

The *source ("Source3_csv.R")* command can be marked and executed. Through this the automated assessment of the Vigi4Eudra-score is carried out fully automatised in the background. The results will be saved in automatically generated data files stored within the file path specified in step 1.

### **Background information source code 1**

##### **Source code 1: 5^th^ step**

Through the source function within step number 5, the application tests a) whether all necessary source code files are available in the given file path and b) whether all R packages required for subsequent functions are installed and activated. When the application is used for the first time on a computer, Source1 automatically downloads the necessary packages. Depending on individual computer settings, it may be necessary to execute this command two consecutive times when it is used for the first time.

### **Background information source code 2**

##### **Source code 2: 7^th^ step**

This command scans the imported line listing for all columns required for the following assessment using the Vigi4Eudra-score. The underlying code is programmed to automatically detect the specific column within the EudraVigilance line listing based on unique keywords or keyword combinations. This allows even line listings with different column sorting compared to the current export structure from EudraVigilance to be used. Furthermore, line listings containing fewer columns than standard line listings can also be processed, insofar as all those required for the evaluation of the Vigi4Eudra-score are available.

## **Export datasets**

The different excel result files contain:

- **ControlTable_Extended:** All information assessed in the Vigi4Eudra-score for each ADR-drug combination of all reports is listed in this table. On the one hand, the exact information contained in the report is specified for each category and, on the other hand, a superordinate assessment is made (_evaluated columns) indicating whether information was available or not.
- **FullCalculation_PerReactionDrug:** All calculated Vigi4Eudra-scores for each ADR-drug combination of a report. Additionally, the presence (value equal to 1) or absence (value equal to penalty of respective category) of each information is listed for each ADR-drug combination of each report
- **EssenceOfCalculation_PerReactionDrug**: All calculated Vigi4Eudra-scores for each ADR-drug combination of each report
- **Vigi4EudraScore_PerCase**: The (mean) Vigi4Eudra-score for each report
- **Range_PerCase**: The number and the values of different Vigi4Eudra-scores calculated for each report. Additionally, the minimum and maximum values of the Vigi4Eudra-score calculated within each report are specified.
