# Supplementary material for: Vigi4Eudra-score: Evaluation of the completeness of spontaneous adverse drug reaction reports in EudraVigilance
Source: PLoS One. 2026 Feb 25;21(2):e0343694. doi: 10.1371/journal.pone.0343694 (PMC12935194; doi:10.1371/journal.pone.0343694)
Supplement: S1 Table — (DOCX) [file pone.0343694.s002.docx]

## S1 Table. Detailed analysis of the distribution of the values of the Vigi4Eudra-score in the four exemplary datasets.

|  | **Q42021 [n=8480]** | **Anaphylaxis [n=5700]** | **KiDSafe I  [n=335]** | **KiDSafe II [n=94]** |
| --- | --- | --- | --- | --- |
| **Observation** | **Statistical key figures for the difference between the automatically calculated values of the Vigi4Eudra-score and the reference values** | | | |
| Mean (+/- sd) | -0.03 [±0.1] | -0.03 [±0.12] | -0.01 [±0.11] | -0.05 [±0.17] |
| Median [IQR] | 0 [-0.06-0] | 0 [-0.05-0] | 0 [0-+0.02] | 0 [-0.13-+0.02] |
| Range | -0.75 - +0.45 | -0.63 - +0.89 | -0.5 - +0.31 | -0.43 - +0.33 |
| Intraclass correlation coefficient [95% confidence interval] | 0.90  [0.87- 0.92] | 0.91  [0.90 - 0.92] | 0.90  [0.88 - 0.92] | 0.76  [0.65 - 0.84] |
| **Differences between the values** | **Number of reports (total and percentual) with an equal or greater absolute difference (automated Vigi4EV-scores - reference)** | | | |
| No difference | 5582 [65.8%] | 3543 [62.2%] | 228 [68.1%] | 42 [44.7%] |
| ≥0.1 | 2898 [34.2%] | 2157 [37.8%] | 107 [31.9%] | 52 [55.3%] |
| ≥0.2 | 1008 [11.9%] | 818 [14.4%] | 43 [12.8%] | 30 [31.9%] |
| ≥0.3 | 446 [5.3%] | 408 [7.2%] | 18 [5.4%] | 18 [19.1%] |
| ≥0.4 | 183 [2.2%] | 152 [2.7%] | 6 [1.8%] | 6 [6.4%] |
| ≥0.5 | 45 [0.5%] | 35 [0.6%] | 2 [0.6%] | 0 [0%] |
| ≥0.6 | 13 [0.2%] | 4 [0.1%] | 0 [0%] | 0 [0%] |
| ≥0.7 | 3 [0%] | 3 [0.1%] | 0 [0%] | 0 [0%] |
| ≥0.8 | 1 [0%] | 2 [0%] | 0 [0%] | 0 [0%] |
| ≥0.9 | 0 [0%] | 1 [0%] | 0 [0%] | 0 [0%] |
| **Difference between the values** | **Numbers (total and percentual) of reports with corresponding difference in automated Vigi4Eudra-scores - reference** | | | |
| -0.8 | 1 [0%] | - | - | - |
| -0.7 | 2 [0%] | - | - | - |
| -0.6 | 13 [0.2%] | 4 [0.1%] | - | - |
| -0.5 | 29 [0.3%] | 25 [0.4%] | 2 [0.6%] | - |
| -0.4 | 127 [1.5%] | 106 [1.9%] | 4 [1.2%] | 6 [6.4%] |
| -0.3 | 218 [2.6%] | 225 [3.9%] | 6 [1.8%] | 8 [8.5%] |
| -0.2 | 446 [5.3%] | 375 [6.6%] | 17 [5.1%] | 8 [8.5%] |
| -0.1 | 1441 [17%] | 632 [11.1%] | 27 [8.1%] | 12 [12.8%] |
| 0 | 5582 [65.8%] | 3543 [62.2%] | 228 [68.1%] | 42 [44.7%] |
| 0.1 | 449 [5.3%] | 707 [12.4%] | 37 [11%] | 10 [10.6%] |
| 0.2 | 116 [1.4%] | 35 [0.6%] | 8 [2.4%] | 4 [4.3%] |
| 0.3 | 45 [0.5%] | 31 [0.5%] | 6 [1.8%] | 4 [4.3%] |
| 0.4 | 11 [0.1%] | 11 [0.2%] | - | - |
| 0.5 | - | 3 [0.1%] | - | - |
| 0.7 | - | 1 [0%] | - | - |
| 0.8 | - | 1 [0%] | - | - |
| 0.9 | - | 1 [0%] | - | - |
