# Supplementary material for: Vigi4Eudra-score: Evaluation of the completeness of spontaneous adverse drug reaction reports in EudraVigilance
Source: PLoS One. 2026 Feb 25;21(2):e0343694. doi: 10.1371/journal.pone.0343694 (PMC12935194; doi:10.1371/journal.pone.0343694)
Supplement: S2 Table — (DOCX) [file pone.0343694.s003.docx]

## S2 Table. Analyses of reports with differences of ≥ +0.3 or ≤ -0.3 between the values of the Vigi4Eudra-score and the vigiGrade completeness score.

| Differences between the values of the Vigi4Eudra-score and the vigiGrade completeness score | **Categories** | | | | | | | | | **Shares in dataset** | | | | | |
| --- | --- | --- | --- | --- | --- | --- | --- | --- | --- | --- | --- | --- | --- | --- | --- |
|  | **EV Missing** | | **EV Missing - Vigibase just Agegroup** | | | | | **Vigibase Missing** | | **Q4 2021** n^total^=8,480 n^≥+0.3/≤-0.3^=446 | | **Anaphylaxis** n^total^=5,700 n^≥+0.3/≤-0.3^=408 | | **KiDSafe I** n^total^=335 n^≥+0.3/≤-0.3^=18 | |
|  | **Report Type** | **PSQ** | **Country** | **Age** | **Dose (superior)** | **Outcome (superior)** | **Sex** | **Indication (superior)** | **TTO (superior)** | **Total** | **Sum** | **Total** | **Sum** | **Total** | **Sum** |
| Difference ≤-0.3 | No differences found on superior level for these reports | | | | | | | | | 64 [14.3%] | | 183 [44.9%] | | 3 [16.7%] | |
|  | No differences found for categories report type, primary source qualification and country | | |  |  |  |  |  |  | 4 [0.9%] | **326 [73.1%]** | 1 [0.2%] | **177 [43.4%]** | - | **9 [50%]** |
|  |  |  |  |  |  |  |  |  |  | 1 [0.2%] |  | 3 [0.7%] |  | - |  |
|  |  |  |  |  |  |  |  |  |  | 2 [0.4%] |  | 2 [0.5%] |  | - |  |
|  |  |  |  |  |  |  |  |  |  | 1 [0.2%] |  | - |  | - |  |
|  |  |  |  |  |  |  |  |  |  | 38 [8.5%] |  | 5 [1.2%] |  | - |  |
|  |  |  |  |  |  |  |  |  |  | 6 [1.3%] |  | 1 [0.2%] |  | - |  |
|  |  |  |  |  |  |  |  |  |  | 17 [3.8%] |  | 14 [3.4%] |  | - |  |
|  |  |  |  |  |  |  |  |  |  | 13 [2.9%] |  | 5 [1.2%] |  | - |  |
|  |  |  |  |  |  |  |  |  |  | 7 [1.6%] |  | 2 [0.5%] |  | - |  |
|  |  |  |  |  |  |  |  |  |  | 3 [0.7%] |  | 1 [0.2%] |  | - |  |
|  |  |  |  |  |  |  |  |  |  | 5 [1.1%] |  | 1 [0.2%] |  | - |  |
|  |  |  |  |  |  |  |  |  |  | 9 [2%] |  | - |  | - |  |
|  |  |  |  |  |  |  |  |  |  | - |  | 2 [0.5%] |  | - |  |
|  |  |  |  |  |  |  |  |  |  | - |  | 1 [0.2%] |  | - |  |
|  |  |  |  |  |  |  |  |  |  | 1 [0.2%] |  | 1 [0.2%] |  | - |  |
|  |  |  |  |  |  |  |  |  |  | - |  | 1 [0.2%] |  | - |  |
|  |  |  |  |  |  |  |  |  |  | - |  | 1 [0.2%] |  | - |  |
|  |  |  |  |  |  |  |  |  |  | - |  | 2 [0.5%] |  | - |  |
|  |  |  |  |  |  |  |  |  |  | - |  | 1 [0.2%] |  | - |  |
|  |  |  |  |  |  |  |  |  |  | 1 [0.2%] |  | - |  | - |  |
|  |  |  |  |  |  |  |  |  |  | 8 [1.8%] |  | 1 [0.2%] |  | - |  |
|  |  |  |  |  |  |  |  |  |  | 6 [1.3%] |  | 3 [0.7%] |  | - |  |
|  |  |  |  |  |  |  |  |  |  | 5 [1.1%] |  | 22 [5.4%] |  | 1 [5.6%] |  |
|  |  |  |  |  |  |  |  |  |  | 18 [4%] |  | 14 [3.4%] |  | 1 [5.6%] |  |
|  |  |  |  |  |  |  |  |  |  | 1 [0.2%] |  | - |  | - |  |
|  |  |  |  |  |  |  |  |  |  | - |  | 1 [0.2%] |  | - |  |
|  |  |  |  |  |  |  |  |  |  | 1 [0.2%] |  | - |  | - |  |
|  |  |  |  |  |  |  |  |  |  | 3 [0.7%] |  | 2 [0.5%] |  | - |  |
|  |  |  |  |  |  |  |  |  |  | 41 [9.2%] |  | 18 [4.4%] |  | - |  |
|  |  |  |  |  |  |  |  |  |  | 16 [3.6%] |  | 6 [1.5%] |  | - |  |
|  |  |  |  |  |  |  |  |  |  | 89 [20%] |  | 53 [13%] |  | 4 [22.2%] |  |
|  |  |  |  |  |  |  |  |  |  | 4 [0.9%] |  | 2 [0.5%] |  | 1 [5.6%] |  |
|  |  |  |  |  |  |  |  |  |  | 3 [0.7%] |  | - |  | - |  |
|  |  |  |  |  |  |  |  |  |  | 12 [2.7%] |  | 8 [2%] |  | 2 [11.1%] |  |
|  |  |  |  |  |  |  |  |  |  | 11 [2.5%] |  | 3 [0.7%] |  | - |  |
| Difference ≤0.3 | No differences found on ADR report level | | | | | | | | | 40 [9%] | | 9 [2.2%] | | 1 [5.6%] | |
|  | No differences found for categories report type, primary source qualification and country | | |  |  |  |  |  |  | - | **16 [3.6%]** | 4 [1%] | **39 [9.6%** | - | **5 [27.8%]** |
|  |  |  |  |  |  |  |  |  |  | - |  | 1 [0.2%] |  | - |  |
|  |  |  |  |  |  |  |  |  |  | - |  | 1 [0.2%] |  | - |  |
|  |  |  |  |  |  |  |  |  |  | - |  | 1 [0.2%] |  | - |  |
|  |  |  |  |  |  |  |  |  |  | - |  | 1 [0.2%] |  | - |  |
|  |  |  |  |  |  |  |  |  |  | - |  | 1 [0.2%] |  | 1 [5.6%] |  |
|  |  |  |  |  |  |  |  |  |  | - |  | 1 [0.2%] |  | - |  |
|  |  |  |  |  |  |  |  |  |  | 4 [0.9%] |  | 26 [6.4%] |  | 3 [16.7%] |  |
|  |  |  |  |  |  |  |  |  |  | - |  | - |  | 1 [5.6%] |  |
|  |  |  |  |  |  |  |  |  |  | 5 [1.1%] |  | 2 [0.5%] |  | - |  |
|  |  |  |  |  |  |  |  |  |  | 3 [0.7%] |  | - |  | - |  |
|  |  |  |  |  |  |  |  |  |  | - |  | 1 [0.2%] |  | - |  |
|  |  |  |  |  |  |  |  |  |  | 4 [0.9%] |  | - |  | - |  |
