# Supplementary material for: Vigi4Eudra-score: Evaluation of the completeness of spontaneous adverse drug reaction reports in EudraVigilance
Source: PLoS One. 2026 Feb 25;21(2):e0343694. doi: 10.1371/journal.pone.0343694 (PMC12935194; doi:10.1371/journal.pone.0343694)
Supplement: S3 Table — (DOCX) [file pone.0343694.s004.docx]

## S3 Table. Discrepancies on the ADR report level.

| **Category where discrepancies were found** | **Difference** | **Dataset** | | |
| --- | --- | --- | --- | --- |
|  |  | **Q42021** n^total^=8,480 n^≥+0.3/≤-0.3^=446 | **Anaphylaxis** n^total^=5,700 n^≥+0.3/≤-0.3^=408 | **KiDSafe I** n^total^=335 n^≥+0.3/≤-0.3^=18 |
| Age of patient | Combined (Total) | 112 [25.1%] | 80 [19.6%] | 6 [33.3%] |
|  | ≤-0.3 | 108 [24.2%] | 44 [10.8%] | - |
|  | ≥0.3 | 4 [0.9%] | 36 [8.8%] | 6 [33.3%] |
| Dose of drug  (evaluated as present  or not present for all drugs of a report) | Combined (Total) | 56 [12.6%] | 63 [15.4%] | 2 [11.1%] |
|  | ≤-0.3 | 51 [11.4%] | 53 [13%] | 2 [11.1%] |
|  | ≥0.3 | 5 [1.1%] | 10 [2.5%] | - |
| Further Evaluation | Combined (Total) | 104 [23.3%] | 192 [47.1%] | 4 [22.2%] |
|  | Multiples present* | 41 [9.2%] | 147 [36%] | 4 [22.2%] |
|  | No explanation detectable | 63 [14.1%] | 45 [11%] | - |
| Indication of drug (evaluated as present  or not present for all drugs of a report) | Combined (Total) | 142 [31.8%] | 48 [11.8%] | 2 [11.1%] |
|  | ≤-0.3 | 139 [31.2%] | 45 [11%] | 1 [5.6%] |
|  | ≥0.3 | 3 [0.7%] | 3 [0.7%] | 1 [5.6%] |
| Outcome of reaction (evaluated as present  or not present for all reactions of a report) | Combined (Total) | 66 [14.8%] | 21 [5.1%] | 4 [22.2%] |
|  | ≤-0.3 | 62 [13.9%] | 21 [5.1%] | 3 [16.7%] |
|  | ≥0.3 | 4 [0.9%] | - | 1 [5.6%] |
| Sex of patient | Combined (Total) | - | 2 [0.5%] | - |
|  | ≤-0.3 | - | 1 [0.2%] | - |
|  | ≥0.3 | - | 1 [0.2%] | - |
| Time to onset (evaluated as present  or not present for all drug-reaction combinations of a report) | Combined (Total) | 182 [40.8%] | 99 [24.3%] | 5 [27.8%] |
|  | ≤-0.3 | 182 [40.8%] | 94 [23%] | 5 [27.8%] |
|  | ≥0.3 | - | 5 [1.2%] | - |
| * multiples present= drug/reaction specific information (e.g. dose, time to onset) were present for some drugs/reactions, but not all of them | | | | |
