# Supplementary material for: Vigi4Eudra-score: Evaluation of the completeness of spontaneous adverse drug reaction reports in EudraVigilance
Source: PLoS One. 2026 Feb 25;21(2):e0343694. doi: 10.1371/journal.pone.0343694 (PMC12935194; doi:10.1371/journal.pone.0343694)
Supplement: S4 Table — (DOCX) [file pone.0343694.s005.docx]

## S4 Table. Discrepancies on the ADR-drug level.

| **Category in which multiples were found** | **Datasource in which multiples were found** | **Q4 2021** n^total^=8,480 n^≥+0.3/≤-0.3^=446 | **Anaphylaxis** n^total^=5,700 n^≥+0.3/≤-0.3^=408 | **KiDSafe I** n^total^=335 n^≥+0.3/≤-0.3^=18 |
| --- | --- | --- | --- | --- |
| Difference ≥+0.3 | | | | |
| Doses | EudraVigilance (EV) | - | - | - |
|  | VigiBase | 7 [17.1%] | 1 [0.7%] | - |
|  | For same report in EV and VigiBase | - | - | - |
| Indications | EV | - | - | - |
|  | VigiBase | 4 [9.8%] | - | - |
|  | For same report in EV and VigiBase | - | - | - |
| Outcomes | EV | 3 [7.3%] | 1 [0.7%] | 1 [25%] |
|  | VigiBase | 3 [7.3%] | - | 1 [25%] |
|  | For same report in EV and VigiBase | 3 [7.3%] | - | 1 [25%] |
| Time to onsets | EV | - | - | - |
|  | VigiBase | - | - | - |
|  | For same report in EV and VigiBase | - | - | - |
| Difference ≤-0.3 | | | | |
| Doses | EV | 2 [4.9%] | 27 [18.4%] | 2 [50%] |
|  | VigiBase | 10 [24.4%] | 65 [44.2%] | 3 [75%] |
|  | For same report in EV and VigiBase | 2 [4.9%] | 27 [18.4%] | 2 [50%] |
| Indications | EV | 3 [7.3%] | 2 [1.4%] | 2 [50%] |
|  | VigiBase | 9 [22%] | 37 [25.2%] | 1 [25%] |
|  | For same report in EV and VigiBase | 3 [7.3%] | 2 [1.4%] | 1 [25%] |
| Outcomes | EV | 15 [36.6%] | 59 [40.1%] | 3 [75%] |
|  | VigiBase | 15 [36.6%] | - | 3 [75%] |
|  | For same report in EV and VigiBase | 15 [36.6%] | - | 3 [75%] |
| Time to onsets | EV | 24 [58.5%] | 138 [93.9%] | 3 [75%] |
|  | WHO | - | - | - |
|  | For same report in EV and VigiBase | - | - | - |
