# Supplementary material for: Vigi4Eudra-score: Evaluation of the completeness of spontaneous adverse drug reaction reports in EudraVigilance
Source: PLoS One. 2026 Feb 25;21(2):e0343694. doi: 10.1371/journal.pone.0343694 (PMC12935194; doi:10.1371/journal.pone.0343694)
Supplement: S5 Table — (DOCX) [file pone.0343694.s006.docx]

## S5 Table. Comparison mean value and best value of the Vigi4Eudra-score to the individual assessment.

| **Observation** | **Comparison mean value of the Vigi4Eudra-score vs. individual assessment** | **Comparison best value of the Vigi4Eudra-score (highest value) vs. individual assessment** |
| --- | --- | --- |
| **KiDSafe II [n=94]** | | |
| Mean (+/- SD) | -0.05 [±0.17] | -0.01 [±0.16] |
| Median [IQR] | 0 [-0.13-0.02] | 0 [-0.04 - 0.05] |
| Range | -0.43 - 0.333 | -0.41 - 0.37 |
| Intraclass correlation coefficient [95% confidence interval] | 0.76  [0.65 - 0.84] | 0.82  [0.75 - 0.88] |
| **Absolute difference of values** | **Number of reports (total and percentual) per calculated difference** | |
| ±0 | 42 [44.7%] | 54 [57.4%] |
| ≤0.1/≥-0.1 | 64 [68.1%] | 74 [78.7%] |
| ≤0.2/≥-0.2 | 76 [80.9%] | 78 [83.0%] |
| ≤0.3/≥-0.3 | 88 [93.7%] | 87 [92.6%] |
| ≤0.4/≥-0.4 | 94 [100%] | 94 [100%] |
| **Difference of values** | **Numbers (total and percentual) of difference of values** | |
| -0.5 | - | - |
| -0.4 | 6 [6.4%] | 6 [6.4%] |
| -0.3 | 8 [8.5%] | 4 [4.3%] |
| -0.2 | 8 [8.5%] | 2 [2.1%] |
| -0.1 | 12 [12.8%] | 5 [5.3%] |
| 0 | 42 [44.7%] | 54 [57.4%] |
| 0.1 | 10 [10.6%] | 15 [16%] |
| 0.2 | 4 [4.3%] | 2 [2.1%] |
| 0.3 | 4 [4.3%] | 5 [5.3%] |
| 0.4 | - | 1 [1.1%] |
| 0.5 | - | - |
