# Supplementary material for: Vigi4Eudra-score: Evaluation of the completeness of spontaneous adverse drug reaction reports in EudraVigilance
Source: PLoS One. 2026 Feb 25;21(2):e0343694. doi: 10.1371/journal.pone.0343694 (PMC12935194; doi:10.1371/journal.pone.0343694)
Supplement: S1 Fig — (DOCX) [file pone.0343694.s007.docx]

## S1 Fig. Differences identified in the information provided per category.


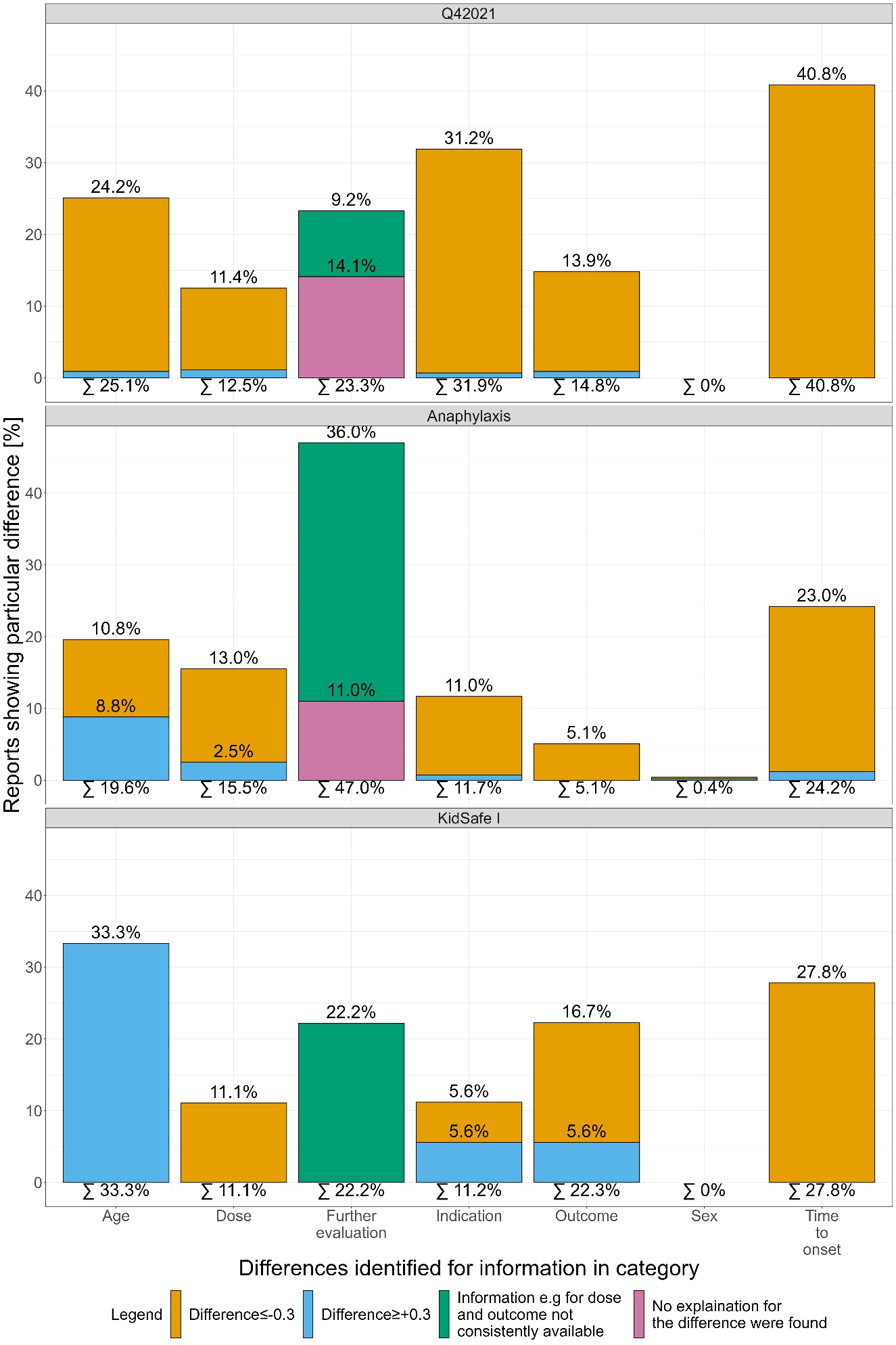


Legend S1 Fig: S1 Fig displays the share of reports for which a certain potential explanation for larger differences between the vigi4Eudra-score and the vigiGrade completeness score were found. Percentual shares were calculated based on the sum of the number of reports in each dataset showing a difference of ≤ -0.3 or ≥ +0.3. Reports with differences equal or smaller than -0.3 are coloured in blue. Reports with differences greater or equal to +0.3 are shown in orange. As soon as differences in the overall absence or presence of information in both datasets in the categories age, dose, indication, outcome, sex and/or time to onset were detected they were listed in the respective categories. An example for this evaluation would be: *no outcomes were reported for all* reactions of the ADR report extracted from EudraVigilance whereas *outcomes for all* reactions were present for this exact ADR report extracted from VigiBase. For those reports where no differences were found on this superior ADR report level, further investigations regarding differences within the categories on the ADR-drug level were carried out. An example for this evaluation would be: *no outcomes were reported for all reactions* of the ADR report extracted from EudraVigilance whereas *outcomes for a few* reactions were present for this exact ADR report extracted from VigiBase. If potential explanations were found on this level, they are represented in green in the category “further evaluation” on the x-axis. The number of ADR reports for which no explanation was found is depicted in purple.
