# Supplementary material for: Vigi4Eudra-score: Evaluation of the completeness of spontaneous adverse drug reaction reports in EudraVigilance
Source: PLoS One. 2026 Feb 25;21(2):e0343694. doi: 10.1371/journal.pone.0343694 (PMC12935194; doi:10.1371/journal.pone.0343694)
Supplement: S1 File — (DOCX) [file pone.0343694.s008.docx]

**# Please copy the following R code in your RStudio code editor.**

# (Sourcecode based) Version 1.0 //// xlsx //// April 2024

#

# Welcome to the automated assessment of the Vigi4Eudra score

#

# This application uses different source codes containing

# all necessary functions

#

# You only need to carry out the following few steps outlined in this file

# starting in code-line: 37.

#

#

# If you have any questions, please contact:

#

# Patrick Christ or Dr. Diana Dubrall

# Kurt-Georg-Kiesinger-Allee 3 # Kurt-Georg-Kiesinger-Allee 3

# 53175 Bonn # 53175 Bonn

# Mail: Patrick.Christ(at)bfarm.de # Mail: Diana.Dubrall (at)bfarm.de

# Phone: +49 228 99 307 4361 # Phone: +49 228 99 307 5345

#

#

# Disclaimer

# The Vigi4Eudra Score was created with the utmost diligence. The application

# processes and analyses line listings of adverse drug reaction (ADR) reports

# provided by the the European ADR database EudraVigilance of the

# European Medicines Agency (EMA). However, we cannot guarantee the accuracy,

# completeness, usability and actuality of these data or the results derived

# from them. Except in the case of intentional misconduct, liability for any

# damages and consequential damages arising from the use of information,

# knowledge, results obtained, materials and documents, prior knowledge and old

# property rights is excluded. This application/code is used on your own

# responsibility and at your own risk.

#

# Please note that by using this application/code you accept the disclaimer,

# otherwise please refrain from using it.

################################################################################

#Start

# Removal of old data in the global environment

rm(list=ls())

################################################################################

################################################################################

################################################################################

################################################################################

################################################################################

#Part 1: Defining the file path:

setwd("B:/Example/Example/Example/Example")

source("Source1_xlsx.R")

# Sourcecode 1 downloads and activates the required R-Studio packages

# in the background.

# This code must be executed every time the application is used.

################################################################################

################################################################################

################################################################################

################################################################################

################################################################################

# Part 2: Importing the relevant dataset (line listing from EudraVigilance)

Dataframe1<-read_excel("Example.xlsx")

source("Source2_xlsx.R")

################################################################################

################################################################################

################################################################################

################################################################################

################################################################################

# Part 3: Processing of the automated Vigi4Eudra score assessment

#

# Please notice: All cases whose case report number occurs more than once in the

# given line listing will be further and separately processed. Later on they can

# be differentiated based on their different "safety report identifiers"

# In all result files a designated column will warn you for potential duplicates.

# In this colloumn all "safety report identifiers" for multiply listed

# "Case repot numbers" will be given.

# Your preferred short name, which you can enter in this step and according to

# which all result files will be labelled, should be as specific as possible

# for your data set, but also as short as possible. We recommend, for example,

# naming a characteristic of the dataset in combination with the respective date.

# e.g "20230527_AllergicReactions" for an dataset including allergic reactions

# which was processed on the 27th may 2023.

# Please enter a preferred shortname describing your dataset.

# All documents generated by this application will contain your specified name

# together with a general description of the content

Dataframename<-paste("Designated_Name_For_Resultsfiles")

source("Source3_xlsx.R")
