# Supplementary material for: Vigi4Eudra-score: Evaluation of the completeness of spontaneous adverse drug reaction reports in EudraVigilance
Source: PLoS One. 2026 Feb 25;21(2):e0343694. doi: 10.1371/journal.pone.0343694 (PMC12935194; doi:10.1371/journal.pone.0343694)
Supplement: S2 File — (DOCX) [file pone.0343694.s009.docx]

**# Please copy the following R code in your RStudio code editor and save it as “Source1_xlsx” in the same folder as your dataset.**

################################################################################

################################################################################

################################################################################

######################## ##############################

##################### ###########################

################# ########################

############# ####################

########## ############################################ ###############

########## ############################################### ##############

########## #### ### #### #### #### ##############

########## #### ### #### #### #### ##############

########## #### ############ ######## ###### #### ##### #### ##############

########## #### ############ ######## ###### #### ##### #### ##############

########## #### ############ ######## ###### #### ##### #### ##############

########## #### ############ ######## ###### #### ##### #### ##############

########## #### ####### ######## ###### #### #### ##############

########## #### ####### ######## ###### #### #### ##############

########## ######### ####### ######## ###### #### ########## ##############

########## ######### ####### ######## ###### #### ########## ##############

########## ######### ####### ######## ###### #### ########## ##############

########## ######### ####### ######## ###### #### ########## ##############

########## #### ####### ######## #### ########## ##############

########## #### ####### ######## #### ########## ##############

########## ################################################# ##############

########## ############################################## ##############

################# ########################

##################### ###########################

##################### ###########################

######################## ##############################

################################################################################

################################################################################

################################################################################

################################################################################

# Please do not change anything in the script below!!!!

# All required steps have to be carried out exclusively in the user file.

#Sourcecode 1:

# 1. Download and activation of required R-studio packages

if (!require(dplyr)) install.packages('dplyr')

if (!require(tidyr)) install.packages('tidyr')

if (!require(stringr)) install.packages('stringr')

if (!require(lubridate)) install.packages('lubridate')

if (!require(purrr)) install.packages('purrr')

if (!require(readxl)) install.packages('readxl')

library("dplyr")

library("tidyr")

library("stringr")

library("lubridate")

library("purrr")

library("readxl")

ifelse(file.exists("Source1_xlsx.R") & file.exists("Source2_xlsx.R") & file.exists("Source3_xlsx.R"),

print("Excellent, all the source codes required are available in your specified file path. All packages required for the furhter processing were installed and should be activated by now. You can go on!"),

print("The file path you have given in the first step does not contain all required source codes. Please check if you have designated the right file path and if it contains all source codes with their original names (Source1_xlsx(.R);Source2_xlsx(.R);Source3_xlsx(.R). Additionally please check if you have designated the file path by replacing all backslashes by 'normal' slashes "))
