# Supplementary material for: Vigi4Eudra-score: Evaluation of the completeness of spontaneous adverse drug reaction reports in EudraVigilance
Source: PLoS One. 2026 Feb 25;21(2):e0343694. doi: 10.1371/journal.pone.0343694 (PMC12935194; doi:10.1371/journal.pone.0343694)
Supplement: S4 File — (DOCX) [file pone.0343694.s011.docx]

**# Please copy the following R code in your RStudio code editor and save it as “Source3_xlsx” in the same folder as your dataset.**

################################################################################

################################################################################

################################################################################

######################## ##############################

##################### ###########################

################# ########################

############# ####################

########## ############################################ ###############

########## ############################################### ##############

########## #### ### #### #### #### ##############

########## #### ### #### #### #### ##############

########## #### ############ ######## ###### #### ##### #### ##############

########## #### ############ ######## ###### #### ##### #### ##############

########## #### ############ ######## ###### #### ##### #### ##############

########## #### ############ ######## ###### #### ##### #### ##############

########## #### ####### ######## ###### #### #### ##############

########## #### ####### ######## ###### #### #### ##############

########## ######### ####### ######## ###### #### ########## ##############

########## ######### ####### ######## ###### #### ########## ##############

########## ######### ####### ######## ###### #### ########## ##############

########## ######### ####### ######## ###### #### ########## ##############

########## #### ####### ######## #### ########## ##############

########## #### ####### ######## #### ########## ##############

########## ################################################# ##############

########## ############################################## ##############

################# ########################

##################### ###########################

##################### ###########################

######################## ##############################

################################################################################

################################################################################

################################################################################

################################################################################

# Please do not change anything in the script below!!!!

# All required steps have to be carried out exclusively in the user file.

#Sourcecode 3 consists of the following steps

# 1. detection of vaccines

# 2. detection of hyposensitization solutions

# 3. Removal of all cases including at least one vaccination and or

# hyposensitization solutions

# 4. Check for duplicates (according to Case Report Number)

# 5. "pre-processing of relevant coloumns"

# 6. Evaluation of all cases accorind to vigiGrade score

# 7. Creating all export data

################################################################################

################################################################################

################################################################################

################################################################################

################################################################################

# Identification of vaccines

options(warn=-1)

Dataframe1Impfstoffe1 <- Dataframe1%>%

filter(str_detect(Suspect.Interacting.Enhanced.Reported.Drug.List..Drug.Char...Indication.PT....Action.taken.with.drug....Start.Date...Duration...Dose...Route.., "VACCIN|VIRUS|STRAIN|ADSORBED|NEISSER|TETANU|INFLUENZ|DIPHTERI|PERTUSS|HEPATIT|CONJUGA|POLIO|IMPFUN|CALIFORNIA|INACTIVATED|TOZINAMERAN"))

Dataframe1Impfstoffe2 <- Dataframe1%>%

anti_join(Dataframe1Impfstoffe1)%>%

filter(str_detect(Indication.s..PT.of.the.drug.of.interest.as.reported.in.the.ICSR, "Immunisat"))

Dataframe1OhneImpfstoffe<-Dataframe1%>%anti_join(Dataframe1Impfstoffe1)%>%anti_join(Dataframe1Impfstoffe2)

################################################################################

################################################################################

################################################################################

################################################################################

################################################################################

# Identification of hyposensitization solutions

Dataframe1Allergene1 <- Dataframe1OhneImpfstoffe%>%filter(str_detect(Suspect.Interacting.Enhanced.Reported.Drug.List..Drug.Char...Indication.PT....Action.taken.with.drug....Start.Date...Duration...Dose...Route.., "ALLERGENS|PHLEUM|BETULA|ALNUS|CORYLUS|WESPE|VESPULA|PRATENSE|GETREIDE|ROGGEN|ALK-DEPO|BIENEN|WASP|ALK LYO|DACTYL|ALLERGOVIT|ORALAIR|PARETHAL|POACEA|SECALE CER|POLLEN|SUBLIVAC|ALLERGEN EXTRAC|BAUME TOP|DERMATOPHAGO|GRAZAX|VENOMIL|GRASER|ACARIZA|DEPIGOID"))

################################################################################

################################################################################

################################################################################

################################################################################

################################################################################

# Removal of hyposensitation solutions and vaccines

# Creating a "clean dataset" without vaccines and hyposensitization solutions

Dataframe1GesamtCleanVektor<-Dataframe1OhneImpfstoffe%>%

anti_join(Dataframe1Allergene1)%>%

select(Case.Report.Number)%>%

as_vector()

Dataframe1<-Dataframe1%>%filter(Case.Report.Number %in%Dataframe1GesamtCleanVektor)

################################################################################

################################################################################

##################### Detection of duplicates ##############################

################################################################################

################################################################################

Dataframe1_FuerDublettencheck<-Dataframe1%>%

add_row(Case.Report.Number="DummyCaseReport",Safety.Report.Identifier="Dummy1")%>%

add_row(Case.Report.Number="DummyCaseReport",Safety.Report.Identifier="Dummy2")

Dataframe1DupliakteCheck_Vektor<-Dataframe1_FuerDublettencheck%>%

select(Case.Report.Number)%>%

count(Case.Report.Number)%>%

filter(!n=="1")%>%

select(Case.Report.Number)%>%

as_vector()

Dataframe1Dupliakte_Entsprechende_SafetyIdentifier_Vorarbeit<-Dataframe1_FuerDublettencheck%>%

select(Case.Report.Number,Safety.Report.Identifier)%>%

filter(Case.Report.Number%in%Dataframe1DupliakteCheck_Vektor)%>%

arrange(desc(Case.Report.Number))%>%

group_by(Case.Report.Number)%>%

mutate(id = row_number())%>%

pivot_wider(id_cols = Case.Report.Number,values_from =Safety.Report.Identifier,names_from = id )

Dataframe1Dupliakte_Entsprechende_SafetyIdentifier_Vorarbeit2<-Dataframe1Dupliakte_Entsprechende_SafetyIdentifier_Vorarbeit%>%

colnames()%>%

toupper()%>%

as.data.frame()%>%

mutate(COLNameHelfNummer=row_number())%>%

select(COLNameHelfNummer)%>%

max()

Dataframe1Dupliakte_Entsprechende_SafetyIdentifier<-Dataframe1Dupliakte_Entsprechende_SafetyIdentifier_Vorarbeit%>%

unite("SafetyReportIdentifierderDuplikate",2:all_of(Dataframe1Dupliakte_Entsprechende_SafetyIdentifier_Vorarbeit2),sep="<<<<&>>>>")

##################################################################################

##################################################################################

##################################################################################

# Merge of case report number and safety report identifier to enable processing

# of duplicates (by case report number)

Dataframe1<-Dataframe1%>%

select(Case.Report.Number,Safety.Report.Identifier,everything())%>%

unite("Case.Report.Number",Safety.Report.Identifier:Case.Report.Number,sep="<<<<<>>>>>")

################################################################################

################################################################################

################ Selection of relevant coloumns #######################

################################################################################

################################################################################

Dataframe1<-Dataframe1%>%

select(Case.Report.Number,Reaction.List.PT..Outcome...Date...Duration.,Suspect.Interacting.Enhanced.Reported.Drug.List..Drug.Char...Indication.PT....Action.taken.with.drug....Start.Date...Duration...Dose...Route..,Primary.Source.Qualification,Sex,Age,Report.Type,Narrative.Present,Narrative..reporter.s.comments.and.sender.s.comments..first.3000.characters.,Country,Structured.Medical.History..Continuing...Text.,Concomitant.Not.Administered.Enhanced.Reported.Drug.List..Drug.Char...Indication.PT....Action.taken.with.drug....Start.Date...Duration...Dose...Route..,Serious)

################################################################################

################################################################################

########## Separation into two dataframes for better processing #########

################################################################################

################################################################################

Dataframe1.Arzneistoffe<-Dataframe1

Dataframe1.AlleRelevantenAngaben<-Dataframe1%>%

select(!Suspect.Interacting.Enhanced.Reported.Drug.List..Drug.Char...Indication.PT....Action.taken.with.drug....Start.Date...Duration...Dose...Route..)

################################################################################

################################################################################

###################### (Pre-)Processing of drugs #########################

################################################################################

################################################################################

Dataframe1.Arzneistoffe_Bearbeitet<-Dataframe1.Arzneistoffe%>%

select(Case.Report.Number,Suspect.Interacting.Enhanced.Reported.Drug.List..Drug.Char...Indication.PT....Action.taken.with.drug....Start.Date...Duration...Dose...Route..)%>%

separate(Suspect.Interacting.Enhanced.Reported.Drug.List..Drug.Char...Indication.PT....Action.taken.with.drug....Start.Date...Duration...Dose...Route..,into = paste0("SUSAM_",1:150), sep="<BR><BR>", fill="right")%>%

gather("SUSAM identifier", "SUSAM",2:151)%>%

drop_na(SUSAM)%>%

separate(SUSAM, into=c("SUSAM", "MoreInICSRINfoEntfernen"),sep = " - More in ICSR")%>%

select(!MoreInICSRINfoEntfernen)%>%

separate(SUSAM, into=c("SUSAM", "MoreInICSRINfoEntfernen"),sep = " - More in ICSR")%>%

select(!MoreInICSRINfoEntfernen)%>%

separate(SUSAM, into=c("SUSAM_StartDate_Duration_Dose", "Route"),sep = "\\ -(?!.* -)")%>%

separate(Route,into=c("Route","Klammerentfernen"),sep="\\]\\)")%>%

select(!Klammerentfernen)%>%

separate(SUSAM_StartDate_Duration_Dose, into=c("SUSAM_StartDate_Duration", "Dose"),sep = "\\- (?!.*-)")%>%

separate(SUSAM_StartDate_Duration, into=c("SUSAM_StartDate", "Duration"),sep = "\\- (?!.*-)")%>%

separate(SUSAM_StartDate, into=c("SUSAM", "StartDate"),sep="\\[(?=[^\\[]+$)")%>%

separate(SUSAM, into=c("SUSAM", "Bindestrichentfernen"),sep = "\\- (?!.*-)")%>%

select(!Bindestrichentfernen)%>%

separate(SUSAM, into=c("SUSAM", "Action_taken_with_drug"),sep = "\\- (?!.*-)")%>%

separate(SUSAM, into=c("SUSAM", "Indication_PT"),sep = "\\ - (?!.* - )")%>%

separate(SUSAM, into=c("SUSAM", "Klammerentfernen"),sep = " \\((?!.* \\()")%>%

select(!Klammerentfernen)%>%

separate(SUSAM, into=c("SUSAM", "Klammerentfernen"),sep = "]" ,extra="merge")%>%

select(!Klammerentfernen)%>%

mutate(Indication_PT=na_if(Indication_PT,"n/a "))%>%

mutate(Action_taken_with_drug=na_if(Action_taken_with_drug,"n/a "))%>%

mutate(StartDate=na_if(StartDate,"n/a "))%>%

mutate(Duration=na_if(Duration,"n/a "))%>%

mutate(Dose=na_if(Dose,"n/a"))%>%

mutate(Route=na_if(Route," n/a"))%>%

mutate(Indication_PT=na_if(Indication_PT,"Unknown"))%>%

mutate(Indication_PT=na_if(Indication_PT,"Product used for unknown indication "))%>%

mutate(Action_taken_with_drug=na_if(Action_taken_with_drug,"Unknown "))%>%

mutate(StartDate=na_if(StartDate, " Unknown"))%>%

mutate(Duration=na_if(Duration," Unknown"))%>%

mutate(Dose=na_if(Dose," Unknown"))%>%

mutate(Route=na_if(Route," Unknown"))

################################################################################

################################################################################

################## (Pre-)Processing of Reactions ######################

################################################################################

################################################################################

Dataframe1.AlleRelevantenAngaben_Bearbeitet_1<-Dataframe1.AlleRelevantenAngaben%>%

select(Case.Report.Number,Reaction.List.PT..Outcome...Date...Duration.)%>%

separate(Reaction.List.PT..Outcome...Date...Duration.,into = paste0("Reaction_",1:150), sep="<BR><BR>", fill="right")%>%

gather("Reaction text", "Reaction",2:151)%>%

drop_na(Reaction)%>%

separate(Reaction, into = c("Reaction", "Duration"), sep = "\\-(?!.*-)", remove = FALSE)%>%

separate(Duration,into = c("Duration","Klammerentfernen"), sep="\\)", fill="right")%>%

select(!Klammerentfernen)%>%

separate(Reaction, into = c("Reaction", "ReactionDate"), sep = "\\-(?!.*-)", remove = FALSE)%>%

separate(Reaction, into = c("Reaction", "Outcome"), sep = "\\((?!.*-)", remove = FALSE)%>%

mutate(Duration=na_if(Duration," n/a"))%>%

mutate(ReactionDate=na_if(ReactionDate," n/a "))%>%

mutate(Outcome=na_if(Outcome," n/a"))%>%

mutate(Duration=na_if(Duration,"Unknown "))%>%

mutate(ReactionDate=na_if(ReactionDate," Unknown"))%>%

mutate(Outcome=na_if(Outcome,"Unknown "))%>%

select(Case.Report.Number,"Reaction text",Reaction,Duration,ReactionDate,Outcome)

Dataframe1.AlleRelevantenAngaben_Bearbeitet_2<-Dataframe1.AlleRelevantenAngaben%>%

select(!Reaction.List.PT..Outcome...Date...Duration.)%>%

separate(Primary.Source.Qualification, into = c("Primary.Source.Qualification", "PSQ_Detailed"), sep = " \\(", remove = FALSE)%>%

select(!PSQ_Detailed)

Dataframe1.AlleRelevantenAngaben_Bearbeitet<-merge.data.frame(x=Dataframe1.AlleRelevantenAngaben_Bearbeitet_1, y=Dataframe1.AlleRelevantenAngaben_Bearbeitet_2, by = "Case.Report.Number")

################################################################################

################################################################################

######### Recombining both datasets #############

################################################################################

################################################################################

Dataframe1.Auswertungsversion<-merge.data.frame(x=Dataframe1.Arzneistoffe_Bearbeitet, y=Dataframe1.AlleRelevantenAngaben_Bearbeitet, by = "Case.Report.Number")%>%

transform(StartDate=dmy(StartDate))%>%

transform(ReactionDate=dmy(ReactionDate))%>%

mutate('TimeToOnset_days'=paste0(ReactionDate- StartDate))%>%

mutate('TimeToOnset_days'=na_if(TimeToOnset_days,"NA"))%>%

transform(TimeToOnset_days=as.character(TimeToOnset_days))%>%

separate(Narrative.Present,into = c("NarrativePresent","WordCountNarrative"), sep=" \\(", fill="right")%>%

separate("WordCountNarrative",into = c("WordCountNarrative","EntfernteKlammer"), sep="\\ ", fill="right",extra="merge")%>%

select(!EntfernteKlammer)

################################################################################

################################################################################

############### Assignment of Available and not available #################

################################################################################

################################################################################

AutomatisierteDokuqualitaet_Kontrolltabelle<-Dataframe1.Auswertungsversion%>%

separate(SUSAM,into=c("SUSAM","Klammerentfernen"),sep="\\]",fill="right")%>%

select(!Klammerentfernen)%>%

separate(SUSAM,into=c("Markenname","ActiveIngredient"),sep="\\[",fill="right")%>%

mutate(OutcomeBewertet = ifelse(Outcome =="NA",NA, "Available"))%>%

#hier beim Time to Onset kann man eventuell noch einen Sinnvollen Filter einbauen

mutate(TimeToOnsetBewertet = ifelse(TimeToOnset_days%in% c(0:100000),"Available",NA))%>%

mutate(SexBewertet = ifelse(Sex =="Male","Available",ifelse(Sex =="Female","Available", NA)))%>%

mutate(AgeBewertet = ifelse(Age %in% c(0:350),"Available",NA))%>%

mutate(ReportTypeBewertet = ifelse(Report.Type =="NA",NA, "Available"))%>%

mutate(IndicationBewertet = ifelse(Indication_PT =="NA",NA,ifelse(Indication_PT ==" Product used for unknown indication ",NA, "Available")))%>%

mutate(DoseBewertet = ifelse(Dose =="NA",NA, "Available"))%>%

#Beim Narrative k?nnte man noch einen mindest Wordcount einbauen

mutate(NarrativeVorhandenBewertet = ifelse(NarrativePresent =="Yes","Available",NA))%>%

mutate(NarrativeVorhandenBewertet = ifelse(Narrative..reporter.s.comments.and.sender.s.comments..first.3000.characters.=="Narrative - Not available<BR><BR>Reporter Comments- Not available<BR><BR>Sender Comments- Not available",NA,"Available"))%>%

mutate(CountryBewertet= ifelse(Country =="NA",NA, "Available"))%>%

mutate(PrimarySourceQualificationBewertet=ifelse(Primary.Source.Qualification=='Healthcare professional',"Available",ifelse(Primary.Source.Qualification=="Non Healthcare professional","Available",ifelse(Primary.Source.Qualification=="Other","Available",NA))))%>%

mutate(Action_taken_with_drugBewertet = ifelse(Action_taken_with_drug =="NA",NA,"Available"))%>%

select(Case.Report.Number,Markenname,ActiveIngredient,Reaction,SUSAM.identifier,Reaction.text,OutcomeBewertet,Outcome,TimeToOnsetBewertet,TimeToOnset_days,SexBewertet,Sex,AgeBewertet,Age,ReportTypeBewertet,Report.Type,IndicationBewertet,Indication_PT,DoseBewertet,Dose,NarrativeVorhandenBewertet,NarrativePresent,Narrative..reporter.s.comments.and.sender.s.comments..first.3000.characters.,CountryBewertet,Country,PrimarySourceQualificationBewertet,Primary.Source.Qualification,Action_taken_with_drugBewertet,Action_taken_with_drug)

AutomatisierteDokuqualitaet_Kontrolltabelle_print<-AutomatisierteDokuqualitaet_Kontrolltabelle%>%

separate(Case.Report.Number,into = c("Safety.Report.Identifier","Case.Report.Number"),sep="<<<<<>>>>>")%>%

merge.data.frame(y=Dataframe1Dupliakte_Entsprechende_SafetyIdentifier,by="Case.Report.Number",all.x=T)%>%

mutate(Duplicatewarning = ifelse(Case.Report.Number%in%Dataframe1DupliakteCheck_Vektor,paste0("Attention: The case report number of this case was detected more than once in your dataset - these are the given Safety Report Identifiers: ",SafetyReportIdentifierderDuplikate)," "))%>%

select(!SafetyReportIdentifierderDuplikate)%>%

select(Duplicatewarning,everything())%>%

rename(OutcomeEvaluated=OutcomeBewertet)%>%

rename(TimeToOnsetEvaluated=TimeToOnsetBewertet)%>%

rename(SexEvaluated=SexBewertet)%>%

rename(AgeEvaluated=AgeBewertet)%>%

rename(ReportTypeEvaluated=ReportTypeBewertet)%>%

rename(IndicationEvaluated=IndicationBewertet)%>%

rename(DoseEvaluated=DoseBewertet)%>%

rename(NarrativeVorhandenEvaluated=NarrativeVorhandenBewertet)%>%

rename(PrimarySourceQualificationEvaluated=PrimarySourceQualificationBewertet)%>%

rename(Action_taken_with_drugEvaluated=Action_taken_with_drugBewertet)%>%

rename(CountryEvaluated=CountryBewertet)

################################################################################

################################################################################

############## Binary assessment of information #####################

################################################################################

################################################################################

AutomatisierteDokuqualitaet_Berechnung_Vorbereitung<-AutomatisierteDokuqualitaet_Kontrolltabelle%>%

mutate(OutcomeScoreVorarbeit = ifelse(OutcomeBewertet %in% c(NA),2,1))%>%

mutate(TimeToOnsetScoreVorarbeit = ifelse(TimeToOnsetBewertet %in% c(NA),2,1))%>%

mutate(SexScoreVorarbeit = ifelse(SexBewertet %in% c(NA),2,1))%>%

mutate(AgeScoreVorarbeit = ifelse(AgeBewertet %in% c(NA),2,1))%>%

mutate(ReportTypeScoreVorarbeit = ifelse(ReportTypeBewertet %in% c(NA),2,1))%>%

mutate(IndicationScoreVorarbeit = ifelse(IndicationBewertet %in% c(NA),2,1))%>%

mutate(DoseScoreVorarbeit = ifelse(DoseBewertet %in% c(NA),2,1))%>%

mutate(NarrativeScoreVorarbeit = ifelse(NarrativeVorhandenBewertet %in% c(NA),2,1))%>%

mutate(CountryScoreVorarbeit = ifelse(CountryBewertet %in% c(NA),2,1))%>%

mutate(PSQScoreVorarbeit = ifelse(PrimarySourceQualificationBewertet%in% c(NA),2,1))%>%

mutate(Action_taken_ScoreVorarbeit = ifelse(Action_taken_with_drugBewertet %in% c(NA),2,1))%>%

#hier wird zur einfacheren Beurteilung der abh?ngigen Bewertung des Narratives

#bezogen auf das Vorhandensein des TTO und des Outcomes eine zus?tzliche Spalte eingef?gt:

mutate(TtoOutcomeCombined = OutcomeScoreVorarbeit+TimeToOnsetScoreVorarbeit)%>%

select(Case.Report.Number,Markenname,ActiveIngredient,Reaction,SUSAM.identifier,Reaction.text,OutcomeScoreVorarbeit,OutcomeBewertet,Outcome,TimeToOnsetScoreVorarbeit,TimeToOnsetBewertet,TimeToOnset_days,SexScoreVorarbeit,SexBewertet,Sex,AgeScoreVorarbeit,AgeBewertet,Age,ReportTypeScoreVorarbeit,ReportTypeBewertet,Report.Type,IndicationScoreVorarbeit,IndicationBewertet,Indication_PT,DoseScoreVorarbeit,DoseBewertet,Dose,NarrativeScoreVorarbeit,NarrativeVorhandenBewertet,NarrativePresent,CountryScoreVorarbeit,CountryBewertet,Country,PSQScoreVorarbeit,PrimarySourceQualificationBewertet,Primary.Source.Qualification,Action_taken_ScoreVorarbeit,Action_taken_with_drugBewertet,Action_taken_with_drug,TtoOutcomeCombined)

AutomatisierteDokuqualitaet_Berechnung_Vorbereitung_print<-AutomatisierteDokuqualitaet_Berechnung_Vorbereitung%>%

separate(Case.Report.Number,into = c("Safety.Report.Identifier","Case.Report.Number"),sep="<<<<<>>>>>")%>%

merge.data.frame(y=Dataframe1Dupliakte_Entsprechende_SafetyIdentifier,by="Case.Report.Number",all.x=T)%>%

mutate(Duplicatewarning = ifelse(Case.Report.Number%in%Dataframe1DupliakteCheck_Vektor,paste0("Attention: The case report number of this case was detected more than once in your dataset - these are the given Safety Report Identifiers: ",SafetyReportIdentifierderDuplikate)," "))%>%

select(!SafetyReportIdentifierderDuplikate)%>%

select(Duplicatewarning,everything())

################################################################################

################################################################################

############# Assignment of Punishment scores ########################

################################################################################

################################################################################

AutomatisierteDokuqualitaet_Scoreverteilung<-AutomatisierteDokuqualitaet_Berechnung_Vorbereitung%>%

mutate(TimeToOnsetScore = ifelse(TimeToOnsetScoreVorarbeit==1,1,"0.5" ))%>%

transform(TimeToOnsetScore=as.numeric(TimeToOnsetScore))%>%

mutate(IndicationScore = ifelse(IndicationScoreVorarbeit==1,1,"0.7"))%>%

transform(IndicationScore=as.numeric(IndicationScore))%>%

mutate(OutcomeScore = ifelse(OutcomeScoreVorarbeit==1,1,"0.7"))%>%

transform(OutcomeScore=as.numeric(OutcomeScore))%>%

mutate(SexScore = ifelse(SexScoreVorarbeit==1,1,"0.7"))%>%

transform(SexScore=as.numeric(SexScore))%>%

mutate(AgeScore = ifelse(AgeScoreVorarbeit==1,1,"0.7"))%>%

transform(AgeScore=as.numeric(AgeScore))%>%

mutate(DoseScore = ifelse(DoseScoreVorarbeit==1,1,"0.90"))%>%

transform(DoseScore=as.numeric(DoseScore))%>%

mutate(CountryScore = ifelse(CountryScoreVorarbeit==1,1,"0.90"))%>%

transform(CountryScore=as.numeric(CountryScore))%>%

mutate(PSQScore = ifelse(PSQScoreVorarbeit==1,1,"0.90"))%>%

transform(PSQScore=as.numeric(PSQScore))%>%

mutate(ReportTypeScore = ifelse(ReportTypeScoreVorarbeit==1,1,"0.9"))%>%

transform(ReportTypeScore=as.numeric(ReportTypeScore))%>%

mutate(NarrativeScore = ifelse(NarrativeScoreVorarbeit==1,1,"0.9"))%>%

transform(NarrativeScore=as.numeric(NarrativeScore))

AutomatisierteDokuqualitaet_Scoreverteilung_print<-AutomatisierteDokuqualitaet_Scoreverteilung%>%

separate(Case.Report.Number,into = c("Safety.Report.Identifier","Case.Report.Number"),sep="<<<<<>>>>>")%>%

merge.data.frame(y=Dataframe1Dupliakte_Entsprechende_SafetyIdentifier,by="Case.Report.Number",all.x=T)%>%

mutate(Duplicatewarning = ifelse(Case.Report.Number%in%Dataframe1DupliakteCheck_Vektor,paste0("Attention: The case report number of this case was detected more than once in your dataset - these are the given Safety Report Identifiers: ",SafetyReportIdentifierderDuplikate)," "))%>%

select(!SafetyReportIdentifierderDuplikate)%>%

select(Duplicatewarning,everything())

################################################################################

################################################################################

#################### Calculation of the vigiScore ######################

################################################################################

################################################################################

AutomatisierteDokuqualitaet_Vigiscore_Berechnung_Ausfuehrlich<-AutomatisierteDokuqualitaet_Scoreverteilung%>%

select(Case.Report.Number,Markenname,ActiveIngredient,Reaction,SUSAM.identifier,Reaction.text,TimeToOnsetScore,IndicationScore,OutcomeScore,SexScore,AgeScore,DoseScore,CountryScore,PSQScore, ReportTypeScore,NarrativeScore)%>%

mutate(VigiScore= paste0(1*TimeToOnsetScore*IndicationScore*OutcomeScore*SexScore*AgeScore*DoseScore*CountryScore*PSQScore*ReportTypeScore*NarrativeScore))%>%

transform(VigiScore=as.numeric(VigiScore))

AutomatisierteDokuqualitaet_Vigiscore_Berechnung_Ausfuehrlich_print<-AutomatisierteDokuqualitaet_Vigiscore_Berechnung_Ausfuehrlich%>%

separate(Case.Report.Number,into = c("Safety.Report.Identifier","Case.Report.Number"),sep="<<<<<>>>>>")%>%

merge.data.frame(y=Dataframe1Dupliakte_Entsprechende_SafetyIdentifier,by="Case.Report.Number",all.x=T)%>%

mutate(Duplicatewarning = ifelse(Case.Report.Number%in%Dataframe1DupliakteCheck_Vektor,paste0("Attention: The case report number of this case was detected more than once in your dataset - these are the given Safety Report Identifiers: ",SafetyReportIdentifierderDuplikate)," "))%>%

select(!SafetyReportIdentifierderDuplikate)%>%

select(Duplicatewarning,everything())

################################################################################

################################################################################

################################################################################

################################################################################

################################################################################

#Runtergebrochen auf die wesentlichen Informationen

AutomatisierteDokuqualitaet_Vigiscore_Berechnung_Wesentlich<-AutomatisierteDokuqualitaet_Vigiscore_Berechnung_Ausfuehrlich%>%

select(Case.Report.Number,Markenname,ActiveIngredient,Reaction,SUSAM.identifier,Reaction.text,VigiScore)

AutomatisierteDokuqualitaet_Vigiscore_Berechnung_Wesentlich_print<-AutomatisierteDokuqualitaet_Vigiscore_Berechnung_Wesentlich%>%

separate(Case.Report.Number,into = c("Safety.Report.Identifier","Case.Report.Number"),sep="<<<<<>>>>>")%>%

merge.data.frame(y=Dataframe1Dupliakte_Entsprechende_SafetyIdentifier,by="Case.Report.Number",all.x=T)%>%

mutate(Duplicatewarning = ifelse(Case.Report.Number%in%Dataframe1DupliakteCheck_Vektor,paste0("Attention: The case report number of this case was detected more than once in your dataset - these are the given Safety Report Identifiers: ",SafetyReportIdentifierderDuplikate)," "))%>%

select(!SafetyReportIdentifierderDuplikate)%>%

select(Duplicatewarning,everything())

################################################################################

################################################################################

################################################################################

################################################################################

################################################################################

# Average vigiSCore per case

AutomatisierteDokuqualitaet_Ergebnis_Vigiscore_Zaehler<-AutomatisierteDokuqualitaet_Vigiscore_Berechnung_Wesentlich%>%

select(Case.Report.Number,VigiScore,SUSAM.identifier,Reaction.text,SUSAM.identifier)%>%

count(Case.Report.Number,VigiScore)%>%

mutate(GesamtscoreMalN=paste0(VigiScore*n))%>%

transform(GesamtscoreMalN=as.numeric(GesamtscoreMalN))%>%

mutate(VigiScore=round(VigiScore,14))%>%

select(-n)%>%

pivot_wider(names_from=VigiScore,values_from = GesamtscoreMalN)%>%

mutate(Zaehler = rowSums(select(., !Case.Report.Number),na.rm = T))%>%

select(Case.Report.Number,Zaehler)

AutomatisierteDokuqualitaet_Ergebnis_Vigiscore_Nenner<-AutomatisierteDokuqualitaet_Vigiscore_Berechnung_Wesentlich%>%

select(Case.Report.Number,VigiScore,SUSAM.identifier,Reaction.text,SUSAM.identifier)%>%

count(Case.Report.Number,VigiScore)%>%

mutate(VigiScore=round(VigiScore,14))%>%

pivot_wider(names_from=VigiScore,values_from = n)%>%

mutate(Nenner = rowSums(select(., !Case.Report.Number),na.rm = T))%>%

select(Case.Report.Number,Nenner)

AutomatisierteDokuqualitaet_Ergebnis_Vigiscore_Gesamtscore<-AutomatisierteDokuqualitaet_Ergebnis_Vigiscore_Zaehler%>%

merge.data.frame(y=AutomatisierteDokuqualitaet_Ergebnis_Vigiscore_Nenner, by="Case.Report.Number")%>%

mutate(MeanVigiscore=paste0(Zaehler/Nenner))%>%

transform(MeanVigiscore=as.numeric(MeanVigiscore))%>%

mutate(MeanVigiscore=round(MeanVigiscore,2))%>%

select(Case.Report.Number,MeanVigiscore)

MeanVigiscore_Case_print<-AutomatisierteDokuqualitaet_Ergebnis_Vigiscore_Gesamtscore%>%

separate(Case.Report.Number,into = c("Safety.Report.Identifier","Case.Report.Number"),sep="<<<<<>>>>>")%>%

merge.data.frame(y=Dataframe1Dupliakte_Entsprechende_SafetyIdentifier,by="Case.Report.Number",all.x=T)%>%

mutate(Duplicatewarning = ifelse(Case.Report.Number%in%Dataframe1DupliakteCheck_Vektor,paste0("Attention: The case report number of this case was detected more than once in your dataset - these are the given Safety Report Identifiers: ",SafetyReportIdentifierderDuplikate)," "))%>%

select(!SafetyReportIdentifierderDuplikate)%>%

select(Duplicatewarning,everything())

################################################################################

################################################################################

################################################################################

################################################################################

################################################################################

# All vigiSCores per case

Vigiscore_RangePerCase_I<-AutomatisierteDokuqualitaet_Vigiscore_Berechnung_Wesentlich%>%

select(Case.Report.Number,VigiScore)%>%

unique()%>%

group_by(Case.Report.Number)%>%

arrange(VigiScore)%>%

mutate(CounterHelfer=row_number())%>%

ungroup()%>%

select(Case.Report.Number,VigiScore,CounterHelfer)%>%

pivot_wider(names_from = CounterHelfer,values_from = VigiScore)

Vigiscore_RangePerCase_I_NumberColoumns<-Vigiscore_RangePerCase_I%>%

slice_head(n=1)%>%

t()%>%

as_tibble()%>%

mutate(Helfer=row_number())%>%

mutate(Helfer=as.numeric(Helfer))%>%

select(Helfer)%>%

slice_tail(n=1)%>%

mutate(Helfer=as.numeric(Helfer))

Vigiscore_RangePerCase_II<-AutomatisierteDokuqualitaet_Vigiscore_Berechnung_Wesentlich%>%

select(Case.Report.Number,VigiScore)%>%

unique()%>%

group_by(Case.Report.Number)%>%

arrange(VigiScore)%>%

mutate(CounterHelfer=row_number())%>%

ungroup()%>%

select(Case.Report.Number,VigiScore,CounterHelfer)%>%

select(!VigiScore)%>%

group_by(Case.Report.Number)%>%

arrange(CounterHelfer)%>%

summarise(across(where(is.numeric), max)) %>%

ungroup()%>%

rename(CountDifferentVigiscoresPerCase=CounterHelfer)

Vigiscore_RangePerCase_III<-AutomatisierteDokuqualitaet_Vigiscore_Berechnung_Wesentlich%>%

select(Case.Report.Number,VigiScore)%>%

unique()%>%

group_by(Case.Report.Number)%>%

summarise(across(where(is.numeric), min)) %>%

ungroup()%>%

rename(VigiScore_Minimum=VigiScore)%>%

mutate(VigiScore_Minimum=paste0(round(VigiScore_Minimum,1)))

Vigiscore_RangePerCase_IV<-AutomatisierteDokuqualitaet_Vigiscore_Berechnung_Wesentlich%>%

select(Case.Report.Number,VigiScore)%>%

unique()%>%

group_by(Case.Report.Number)%>%

summarise(across(where(is.numeric), max)) %>%

ungroup()%>%

rename(VigiScore_Maximum=VigiScore)%>%

mutate(VigiScore_Maximum=paste0(round(VigiScore_Maximum,1)))

Vigiscore_RangePerCase<-Vigiscore_RangePerCase_I%>%

unite(AllVigiScoresForEachCase,2:Vigiscore_RangePerCase_I_NumberColoumns$Helfer,sep="<BR><BR>",na.rm=T)%>%

merge.data.frame(Vigiscore_RangePerCase_II,by="Case.Report.Number")%>%

merge.data.frame(Vigiscore_RangePerCase_III,by="Case.Report.Number")%>%

merge.data.frame(Vigiscore_RangePerCase_IV,by="Case.Report.Number")

Vigiscore_RangePerCase_print<-Vigiscore_RangePerCase%>%

separate(Case.Report.Number,into = c("Safety.Report.Identifier","Case.Report.Number"),sep="<<<<<>>>>>")%>%

merge.data.frame(y=Dataframe1Dupliakte_Entsprechende_SafetyIdentifier,by="Case.Report.Number",all.x=T)%>%

mutate(Duplicatewarning = ifelse(Case.Report.Number%in%Dataframe1DupliakteCheck_Vektor,paste0("Attention: The case report number of this case was detected more than once in your dataset - these are the given Safety Report Identifiers: ",SafetyReportIdentifierderDuplikate)," "))%>%

select(!SafetyReportIdentifierderDuplikate)%>%

select(Duplicatewarning,everything())

################################################################################

################################################################################

######################## Renaming #############################

################################################################################

################################################################################

AutomatisierteDokuqualitaet_Kontrolltabelle_print<-AutomatisierteDokuqualitaet_Kontrolltabelle_print%>%

rename(BrandName=Markenname)%>%

rename(NarrativeVPresentEvaluated=NarrativeVorhandenEvaluated)

AutomatisierteDokuqualitaet_Vigiscore_Berechnung_Ausfuehrlich_print<-AutomatisierteDokuqualitaet_Vigiscore_Berechnung_Ausfuehrlich_print%>%

rename(BrandName=Markenname)%>%

rename(Vigi4EudraScore=VigiScore)

AutomatisierteDokuqualitaet_Vigiscore_Berechnung_Wesentlich_print<-AutomatisierteDokuqualitaet_Vigiscore_Berechnung_Wesentlich_print%>%

rename(BrandName=Markenname)%>%

rename(Vigi4EudraScore=VigiScore)

Vigiscore_RangePerCase_print<-Vigiscore_RangePerCase_print%>%

rename(AllVigi4EudraScoreValues_PerReport=AllVigiScoresForEachCase)%>%

rename(CountDifferentVigi4EudraScore_PerReport=CountDifferentVigiscoresPerCase)%>%

rename(Lowest_Vigi4EudraScore_PerReport=VigiScore_Minimum)%>%

rename(Best_Vigi4EudraScore_PerReport=VigiScore_Maximum)

MeanVigiscore_Case_print<-MeanVigiscore_Case_print%>%

rename(Vigi4EudraScore=MeanVigiscore)

################################################################################

################################################################################

######################## Exportdata #############################

################################################################################

################################################################################

write.csv2(AutomatisierteDokuqualitaet_Kontrolltabelle_print,file=paste(Dataframename,"ControlTable_Extended.csv"),row.names=F)

write.csv2(AutomatisierteDokuqualitaet_Vigiscore_Berechnung_Ausfuehrlich_print,file=paste(Dataframename,"FullCalculation_PerReactionDrug.csv"),row.names=F)

write.csv2(AutomatisierteDokuqualitaet_Vigiscore_Berechnung_Wesentlich_print,file=paste(Dataframename,"EssenceOfCalculation_PerReactionDrug.csv"),row.names=F)

write.csv2(Vigiscore_RangePerCase_print,file=paste(Dataframename,"Range_PerCase.csv"),row.names=F)

write.csv2(MeanVigiscore_Case_print,file=paste(Dataframename,"Vigi4EudraScore_PerCase.csv"),row.names=F)

options(warn=0)

print("

################################################################################################################################################################################################################################################################################################################################################################################################################################################################################################################################################################################################################################################################################################################################################################################################################################################################################################################################################################################################################################################################################################################################################################################################################################################################################################################################################################################################################################################################################################################################################################################################################################################################################################################################################################################################################################################################################################################################################################################################################################################################################################################################################################################################################################################################################################################################################################################################################################################################################################################################################################################################################################################################")

print("The application has carried out all required steps. Now you should find a selection of export data in the given file path.")
