# Supplementary material for: Vigi4Eudra-score: Evaluation of the completeness of spontaneous adverse drug reaction reports in EudraVigilance
Source: PLoS One. 2026 Feb 25;21(2):e0343694. doi: 10.1371/journal.pone.0343694 (PMC12935194; doi:10.1371/journal.pone.0343694)
Supplement: S7 File — (DOCX) [file pone.0343694.s014.docx]

**# Please copy the following R code in your RStudio code editor and save it as “Source2_csv” in the same folder as your dataset.**

################################################################################

################################################################################

################################################################################

######################## ##############################

##################### ###########################

################# ########################

############# ####################

########## ############################################ ###############

########## ############################################### ##############

########## #### ### #### #### #### ##############

########## #### ### #### #### #### ##############

########## #### ############ ######## ###### #### ##### #### ##############

########## #### ############ ######## ###### #### ##### #### ##############

########## #### ############ ######## ###### #### ##### #### ##############

########## #### ############ ######## ###### #### ##### #### ##############

########## #### ####### ######## ###### #### #### ##############

########## #### ####### ######## ###### #### #### ##############

########## ######### ####### ######## ###### #### ########## ##############

########## ######### ####### ######## ###### #### ########## ##############

########## ######### ####### ######## ###### #### ########## ##############

########## ######### ####### ######## ###### #### ########## ##############

########## #### ####### ######## #### ########## ##############

########## #### ####### ######## #### ########## ##############

########## ################################################# ##############

########## ############################################## ##############

################# ########################

##################### ###########################

##################### ###########################

######################## ##############################

################################################################################

################################################################################

################################################################################

################################################################################

# Please do not change anything in the script below!!!!

# All required steps have to be carried out exclusively in the user file.

options(warn=-1)

# In this part of the code all coloumn names (which can be changed from time to

# time in the extracted Line Listings from EV) are identified via specific

# catchwords. By the help of these catchwords the position of the respective

# coloumn is automatically identified by the application

colnames<-Dataframe1%>%

colnames()%>%

toupper()%>%

as.data.frame()%>%

mutate(COLNameHelfNummer=row_number())%>%

filter(str_detect(.,"IDENTIFIER|REPORT|INTEREST|REACTION|NARRATIVE|HISTORY|CONCOMITANT|SEX|AGE|ENHANCED|COUNTRY|SERIOUS|INDICATION|ICSR|PRIMARY|QUALIFICATION"))%>%

filter(!str_detect(.,"SERIOUSNESS"))

Safety.Report.IdentifierRenamer<-colnames%>%

filter(str_detect(.,"IDENTIFIER"))%>%

select(COLNameHelfNummer)%>%

as_vector()

CaseReportNumber<-colnames%>%

filter(str_detect(.,"REPORT"))%>%

filter(!str_detect(.,"REPORTED|EV|TYPE|REPORTER"))%>%

select(COLNameHelfNummer)%>%

as_vector()

ReactionListPTOutcomeDateDuration<-colnames%>%

filter(str_detect(.,"REACTION"))%>%

select(COLNameHelfNummer)%>%

as_vector()

SuspectInteractingEnhancedReportedDrugList<-colnames%>%

filter(str_detect(.,"ENHANCED"))%>%

filter(!str_detect(.,"CONCOMITANT"))%>%

select(COLNameHelfNummer)%>%

as_vector()

SEXRenamer<-colnames%>%

filter(str_detect(.,"SEX"))%>%

select(COLNameHelfNummer)%>%

as_vector()

AGERenamer<-colnames%>%

filter(str_detect(.,"AGE"))%>%

select(COLNameHelfNummer)%>%

as_vector()

NarrativeRenamer<-colnames%>%

filter(str_detect(.,"NARRATIVE"))%>%

filter(!str_detect(.,"COMPLETE|PRESENT"))%>%

select(COLNameHelfNummer)%>%

as_vector()

ReportTypeRenamer<-colnames%>%

filter(str_detect(.,"TYPE"))%>%

select(COLNameHelfNummer)%>%

as_vector()

NarrativePresentRenamer<-colnames%>%

filter(str_detect(.,"NARRATIVE"))%>%

filter(str_detect(.,"PRESENT"))%>%

select(COLNameHelfNummer)%>%

as_vector()

CountryRenamer<-colnames%>%

filter(str_detect(.,"COUNTRY"))%>%

select(COLNameHelfNummer)%>%

as_vector()

MedicalHistoryRenamer<-colnames%>%

filter(str_detect(.,"HISTORY"))%>%

filter(str_detect(.,"STRUCTURED"))%>%

select(COLNameHelfNummer)%>%

as_vector()

MedicalHistoryRenamer<-colnames%>%

filter(str_detect(.,"HISTORY"))%>%

filter(str_detect(.,"STRUCTURED"))%>%

select(COLNameHelfNummer)%>%

as_vector()

CONCOMITANTDRUGSRenamer<-colnames%>%

filter(str_detect(.,"CONCOMITANT"))%>%

select(COLNameHelfNummer)%>%

as_vector()

SERIOUSRenamer<-colnames%>%

filter(str_detect(.,"SERIOUS"))%>%

select(COLNameHelfNummer)%>%

as_vector()

IndicationRenamer<-colnames%>%

filter(str_detect(.,"INDICATION"))%>%

filter(str_detect(.,"INTEREST"))%>%

select(COLNameHelfNummer)%>%

as_vector()

ICSRFormRenamer<-colnames%>%

filter(str_detect(.,"ICSR"))%>%

filter(str_detect(.,"FORM"))%>%

select(COLNameHelfNummer)%>%

as_vector()

PSQFormRenamer<-colnames%>%

filter(str_detect(.,"PRIMARY"))%>%

filter(str_detect(.,"SOURCE"))%>%

filter(str_detect(.,"QUALIFICATION"))%>%

select(COLNameHelfNummer)%>%

as_vector()

# Renaming process

Dataframe1<-Dataframe1%>%

rename(Safety.Report.Identifier=Safety.Report.IdentifierRenamer)%>%

rename(Safety.Report.Identifier=Safety.Report.Identifier...COLNameHelfNummer)%>%

rename(Case.Report.Number=CaseReportNumber)%>%

rename(Case.Report.Number=Case.Report.Number...COLNameHelfNummer)%>%

rename(Reaction.List.PT..Outcome...Date...Duration.=ReactionListPTOutcomeDateDuration)%>%

rename(Reaction.List.PT..Outcome...Date...Duration.=Reaction.List.PT..Outcome...Date...Duration....COLNameHelfNummer)%>%

rename(Suspect.Interacting.Enhanced.Reported.Drug.List..Drug.Char...Indication.PT....Action.taken.with.drug....Start.Date...Duration...Dose...Route..=SuspectInteractingEnhancedReportedDrugList)%>%

rename(Suspect.Interacting.Enhanced.Reported.Drug.List..Drug.Char...Indication.PT....Action.taken.with.drug....Start.Date...Duration...Dose...Route..=Suspect.Interacting.Enhanced.Reported.Drug.List..Drug.Char...Indication.PT....Action.taken.with.drug....Start.Date...Duration...Dose...Route.....COLNameHelfNummer)%>%

rename(Sex=SEXRenamer)%>%

rename(Sex=Sex...COLNameHelfNummer)%>%

rename(Age=AGERenamer)%>%

rename(Age=Age...COLNameHelfNummer)%>%

rename(Narrative..reporter.s.comments.and.sender.s.comments..first.3000.characters.=NarrativeRenamer)%>%

rename(Narrative..reporter.s.comments.and.sender.s.comments..first.3000.characters.=Narrative..reporter.s.comments.and.sender.s.comments..first.3000.characters....COLNameHelfNummer)%>%

rename(Report.Type=ReportTypeRenamer)%>%

rename(Report.Type=Report.Type...COLNameHelfNummer)%>%

rename(Narrative.Present=NarrativePresentRenamer)%>%

rename(Narrative.Present=Narrative.Present...COLNameHelfNummer)%>%

rename(Country=CountryRenamer)%>%

rename(Country=Country...COLNameHelfNummer)%>%

rename(Structured.Medical.History..Continuing...Text.=MedicalHistoryRenamer)%>%

rename(Structured.Medical.History..Continuing...Text.=Structured.Medical.History..Continuing...Text....COLNameHelfNummer)%>%

rename(Concomitant.Not.Administered.Enhanced.Reported.Drug.List..Drug.Char...Indication.PT....Action.taken.with.drug....Start.Date...Duration...Dose...Route..=CONCOMITANTDRUGSRenamer)%>%

rename(Concomitant.Not.Administered.Enhanced.Reported.Drug.List..Drug.Char...Indication.PT....Action.taken.with.drug....Start.Date...Duration...Dose...Route..=Concomitant.Not.Administered.Enhanced.Reported.Drug.List..Drug.Char...Indication.PT....Action.taken.with.drug....Start.Date...Duration...Dose...Route.....COLNameHelfNummer)%>%

rename(Serious=SERIOUSRenamer)%>%

rename(Serious=Serious...COLNameHelfNummer)%>%

rename(Indication.s..PT.of.the.drug.of.interest.as.reported.in.the.ICSR=IndicationRenamer)%>%

rename(Indication.s..PT.of.the.drug.of.interest.as.reported.in.the.ICSR=Indication.s..PT.of.the.drug.of.interest.as.reported.in.the.ICSR...COLNameHelfNummer)%>%

rename(Primary.Source.Qualification=PSQFormRenamer)%>%

rename(Primary.Source.Qualification=Primary.Source.Qualification...COLNameHelfNummer)%>%

rename(ICSR.form=ICSRFormRenamer)%>%

rename(ICSR.form=ICSR.form...COLNameHelfNummer)

Inprozesskontrolle_Source2<-Dataframe1%>%

colnames()%>%

toupper()%>%

as.data.frame()%>%

mutate(COLNameHelfNummer=row_number())%>%

select(COLNameHelfNummer)%>%

slice_tail(n=1)

options(warn=0)

ifelse(Inprozesskontrolle_Source2$COLNameHelfNummer>45, print("In process controll successfull. The application was able to identify the right number of coloumns."),

print("Warning: The in process controll revealed that less coloumns than usual were identified. Please check if you are operating a csv file - Otherwise change for the xlsx script. This could as well be related to the line listing you have extracted from EudraVigilance, if you do not have full access rights."))

print("You can go on with step 3!")
